# Estimation of the Vaporization Enthalpies and Vapor Pressures of α-Tocopherol and Δ^9^-Tetrahydrocannabinol via the Use of a Surrogate, Correlation Gas Chromatography, and Synthetic and Retrosynthetic Analysis

**DOI:** 10.3390/molecules29184332

**Published:** 2024-09-12

**Authors:** Carissa Nelson, Christian Fischer-Lodike, James S. Chickos

**Affiliations:** Department of Chemistry and Biochemistry, 1 University Blvd, University of Missouri-St. Louis, St. Louis, MO 63121, USA; nelsoncarissa@hotmail.com (C.N.); cfischerlodike@gmail.com (C.F.-L.)

**Keywords:** vaporization enthalpy, vapor pressure, correlation gas chromatography, α-tocopherol, tetrahydrocannabinol

## Abstract

A protocol is proposed that combines the use of the known properties of a surrogate containing various functional groups together with n-alkanes as standards to evaluate the properties of much larger related substances using correlation gas chromatography. An objective of this work is to develop options that circumvent the lack of appropriate vaporization enthalpy standards that can be used for evaluation of various thermodynamic properties of larger complex molecules using gas chromatography. The surrogate in this case is 2,2,5,7,8-pentamethylchroman-6-ol (PMC) and is used to evaluate the vaporization enthalpies and vapor pressures of α-tocopherol (α-TOC) and Δ^9^-tetrahydrocannabinol (Δ^9^-THC). The results are compared to the available literature data and to estimated properties. Vaporization enthalpies are also evaluated by a proposed method that involves the use of synthetic and retrosynthetic analysis.

## 1. Introduction

Vitamin E refers to a group of antioxidants found in various seed and vegetable oils [1]. Among the most active is α-tocopherol, a common form of the vitamin in the diet. As a lipid soluble oil, the main biological function of α-tocopherol is to serve as an antioxidant disrupting free radical propagation by reactive oxygen species that lead to damage of cell membranes [1]. As a result of difficulties in obtaining pure synthetic samples of α-tocopherol (α-TOC), a viscous oil containing three asymmetric centers, 2,2,5,7,8-pentamethylchroman-6-ol (PMC), has often been used as a surrogate for in vitro studies [2].

Cannabis has been used as a medicinal plant for millennia [3,4]. Tetrahydrocannabinol (Δ^9^-THC), (6a*R*,10a*R*)-7,8,10a-tetrahydro-6,6,9-trimethyl-3-pentyl-6*H*-dibenzo[*b*,*d*]pyran-1-ol), one of the major active ingredients in cannabis, has been a focal point of interest in research since its isolation, characterization, and synthesis in the mid 1960’s [4,5,6,7]. A typical route of ingestion for people using recreational Δ^9^-THC is through inhalation. The drug enters the lungs in the gas phase and presumably attached to material adsorbed on the particulate matter that constitute the smoke. In both cases, the vapor pressure of liquid Δ^9^-THC near body temperatures should play an important role in the transport mechanism. The decriminalization of cannabis use in many states in the United States will likely cause a rise in use and potential consequences associated with intoxication. Consequently, there is an interest in developing methods for monitoring unacceptable blood levels of Δ^9^-THC as is currently conducted for ethanol. Methods that have been used in research include solid-phase methanol extraction followed by LC-MS/MS separation and quantification [8]. Recently, Lovestead and Bruno [9] reported vapor pressures of Δ^9^-THC over the temperature range *T* = (333–414.3) K using a dynamic head space analysis described as a PLOT-cryo-adsorption apparatus. This study focuses on estimating the vaporization enthalpies and vapor pressures of both Δ^9^-THC and α-TOC by combining experimental measurements with synthetic analysis.

**Figure 1:**
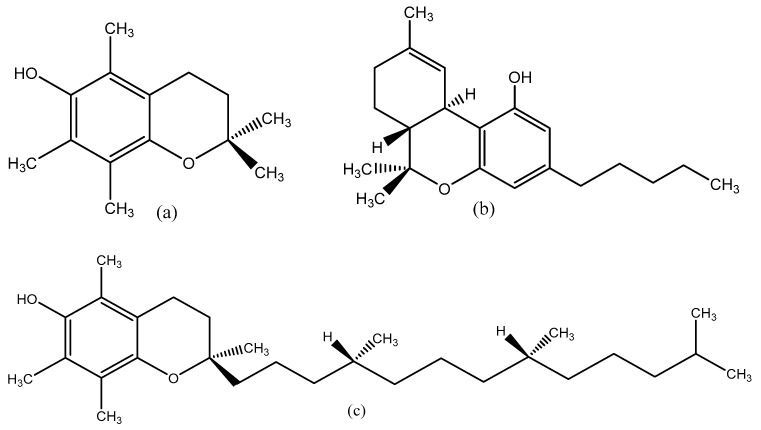
(**a**): 2,2,5,7,8-Pentamethylchroman-6-ol (PMC); (**b**): Δ^9^-tetrahydrocannabinol (Δ^9^-THC); (**c**): α-tocopherol (α-TOC). The structure drawn for α-tocopherol is the *R*, *R*, *R* stereoisomer. The material studied was a racemic mixture of diastereomers.

Despite exhibiting different physiological properties, Δ^9^-THC, α-TOC, and PMC all share some structural similarities including the same two functional groups, a cyclic ether and a phenolic hydroxyl group. The structures of all three substances are illustrated in Figure 1; only one of eight possible stereoisomers of (±) α-TOC is shown, the *R*, *R*, *R* stereoisomer. A number of thermodynamic properties of PMC have been carefully evaluated [2]. This study examines whether the thermodynamic properties of PMC could be useful in evaluating those of the other two substances it structurally and functionally resembles.

Group additivity is a very powerful tool when applied to molecular properties responsive to this technique. Numerous methods have been developed to estimate vaporization enthalpies of organic compounds using bond or group properties. Application generally requires a large number of groups and often other parameters. Two examples of recent group contribution methods for estimating vaporization enthalpy list 125 [10] and 314 [11] groups and parameters. In addition to group values, other parameters are often needed to account for the presence of non-bonded interactions such as steric effects and for hydrogen bonding. While variable in magnitude, it is the non-bonded interactions and hydrogen bonding that are often the most difficult to predict as they appear inclined to ignore group principles. Most estimations via group additivity generally construct the target, group by group. What appears as a simple and useful estimation technique can become complex and prone to miscalculation by users either unfamiliar with the application of the method or overwhelmed by the large number of available groups and possible adjustments.

Current size limitations of many experimental techniques, the lack of appropriate standards, and the low volatility of large molecules has limited the evaluation of some of their thermodynamic properties. The availability of large databases of experimental vaporization enthalpy data of moderately sized molecules is a valuable source of basic information. If properly modified and combined appropriately using group values, they should be able to provide properties of much larger molecules without the need to construct them from fundamental groups [12,13,14,15,16], and in the process, perhaps, also simplifying the estimation and either eliminating or reducing the need to account for other interactions. Depending on the structure of the target and availability of data, it also provides an opportunity for employing alternative synthetic pathways for the estimation. Reproducibility provides some assessment of the quality of the estimation. Recently, such an approach has been used in evaluation of the heat capacities of a series of steroids and other complex molecules [17].

An experimental technique that we have been employing for evaluation of vaporization enthalpies, correlation gas chromatography, while capable of providing evaluations of the vaporization enthalpies of larger molecules, has been impeded by the lack of reliable standards of appropriate size and functionality. With the exception of linear molecules such as n-alkanes and some simple esters and alcohols, there is a lack of sufficiently accurate data on enough related materials containing the appropriate functionalities that could be used reliably as standards. As a means of addressing this problem, the question arose as to whether the vaporization enthalpy of n-alkanes could be used as standards, if the vaporization enthalpy of a surrogate molecule containing the appropriate functionality and structure was experimentally available. The idea is to evaluate both the target and the surrogate via correlation gas chromatography using n-alkanes as standards and then to adjust the resulting vaporization enthalpy of the target using the enthalpy difference obtained between the experimental vaporization enthalpy of the surrogate and the value evaluated using n-alkanes. It seems plausible that for this approach to be applicable, the target should differ from the surrogate only by additional hydrocarbon components.

As a first step in applying this approach, the applicability of synthetic analysis as a means of estimating the vaporization enthalpies of larger molecules needs to be demonstrated. For this application, we have found the group values reported by Guthrie and Taylor to be the most applicable [18]. Unlike other group additivity schemes, it should be noted that the Guthrie and Taylor group method does not appear to distinguish between cyclic and acyclic carbon groups, i.e., cyclic and acyclic methylene, methine, and quaternary carbons. The hydrocarbon group values used in this work are taken from values published by these authors and are reproduced in kJ·mol^−1^ in Table 1. The notation in describing groups used by the authors are basically the descriptors used by Benson [19].

## 2. Vaporization Enthalpies via Synthetic Analysis

### 2.1. n-Alkanes

**Scheme 1:**
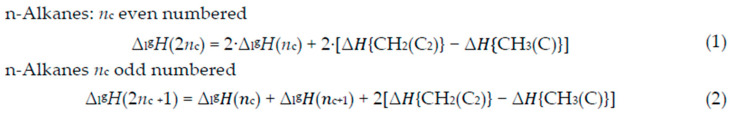
Estimations of Δ_l_^g^*H*(298.15 K) of CH_3_(CH_2_)*_n_c__*-_2_CH_3_ via synthetic analysis (Table 1).

As examples of the applicability of the synthetic analysis approach, several simple estimations are illustrated in the tables and schemes provided below. The vaporization enthalpies of a series of n-alkanes from n-pentane to n-nonane have been used to evaluate the vaporization enthalpies of n-decane to n-eicosane. Also included in Table 2 are estimations of the values for n-heneicosane to n-tetracontane. The vaporization enthalpies of n-pentane to n-eicosane are those recommended by Ruzicka et al. [20]. Equations (1) and (2), used to estimate the vaporization enthalpies of the even and odd n-alkanes via synthetic analysis, are provided in Scheme 1. The term *n*_c_ refers to the number of carbons of the n-alkane. The even numbered n-alkanes in column 4 of Table 2 were calculated using Equation (1), the value in column 2, and the appropriate group values from Table 1. Similarly, those with an odd number of carbons were evaluated using Equation (2) together with the appropriate values from both column 3 and Table 1. Results are illustrated in Table 2. Good agreement is observed for all estimations. The results for n-heneicosane to n-tetracontane are particularly significant since these values were evaluated via an extrapolation process using correlation gas chromatography and recommended values of several smaller n-alkanes [21,22,23].

The following figures and schemes illustrate the use of synthetic analysis to estimate vaporization enthalpies of a series of polycyclic hydrocarbons. For substances containing quaternary carbon atoms, estimations are generated using Equation (3),

Δ_l_^g^*H*(298 K)/kJ.mol^−1^ = 4.69.(*n*_C_ − *n*_Q_) + 1.3.*n*_Q_ + 3.0 (3)

where *n*_C_ and *n*_Q_ refer to the total number of carbons and quaternary carbons, respectively [24]. Some simple examples include the evaluation of the vaporization enthalpy of adamantane and diamantane and their methyl derivatives. Values for only the former are available. More complex systems include the evaluation of the vaporization enthalpies of androstane and cholestane. Experimental values of a few materials used as synthons in the synthetic schemes needed to be adjusted to *T* = 298.15 K. Additional details regarding these adjustments are provided in the Supporting Material (SM). These entries can be identified in the text by the suffix (S; X), where X specifies the Scheme in the Supporting Material (SM).

### 2.2. Synthetic Analysis of Adamantane and Diamantane at T = 298.15 K by Way of Their 2-Methyl Derivatives

**Scheme 2:**
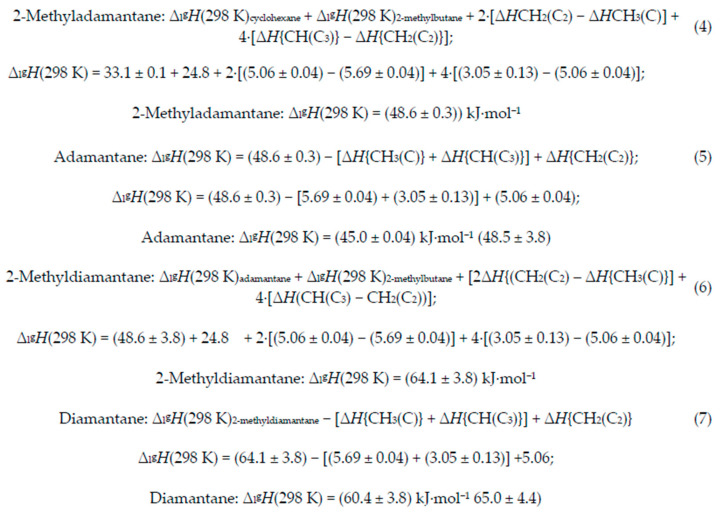
Synthetic analysis of adamantane and diamantane by way of their 1-methyl derivatives [25,26,27].

**Figure 2:**
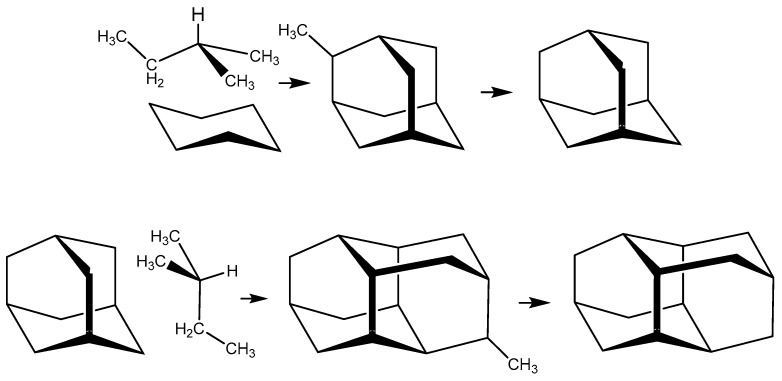
Synthetic analysis of methyladamantane and diamantane by way of 2-methyladamantane and 2-methyldiamantane.

Analysis of both adamantane and diamantane require vaporization enthalpies of both cyclohexane and isobutane. Since *T* = 298.15 K is above the normal boiling temperature of isobutane, the vaporization enthalpy of 2-methylbutane was used in its place. A vaporization enthalpy of 33.1 kJ·mol^−1^ at *T* = 298.15 K has been reported for cyclohexane [25], and 24.8 kJ·mol^−1^ has been reported for 2-methylbutane [26]. Synthetic analysis of both 2-methyladamantane and 2-methyldiamantane, illustrated in Figure 2, are summarized in Scheme 2, Equations (4) and (6). Experimental values are used for all starting materials. Both are solids at *T* = 298.15 K. In the formation of 1-methyladamantane, four methylene groups are converted to methine groups, three methylene groups on the cyclohexane and one on 2-methylbutane; in addition, two methyl groups are converted to methylene groups. In the second step, removal of a methyl group results in a loss of a CH_3_(C) and a CH(C_3_) and formation of a CH_2_(C_2_); Equations (5) and (7) summarize this transformation. The vaporization enthalpies of both adamantane and diamantane could also be synthesized more directly using isobutane. Our preference has been to use vaporization enthalpies of synthons that are liquids at *T* = 298.15 K whenever possible.

### 2.3. Synthetic Analysis of Androstane and Cholestane

**Figure 3:**
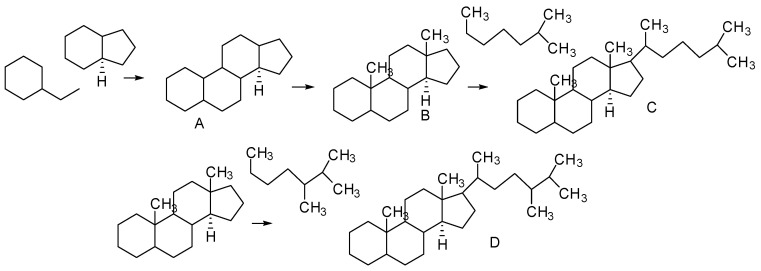
Synthetic analysis of (**A**) “gonane”, (**B**) “androstane”, (**C**) “cholestane”, and (**D**) “ergostane”.

**Scheme 3:**
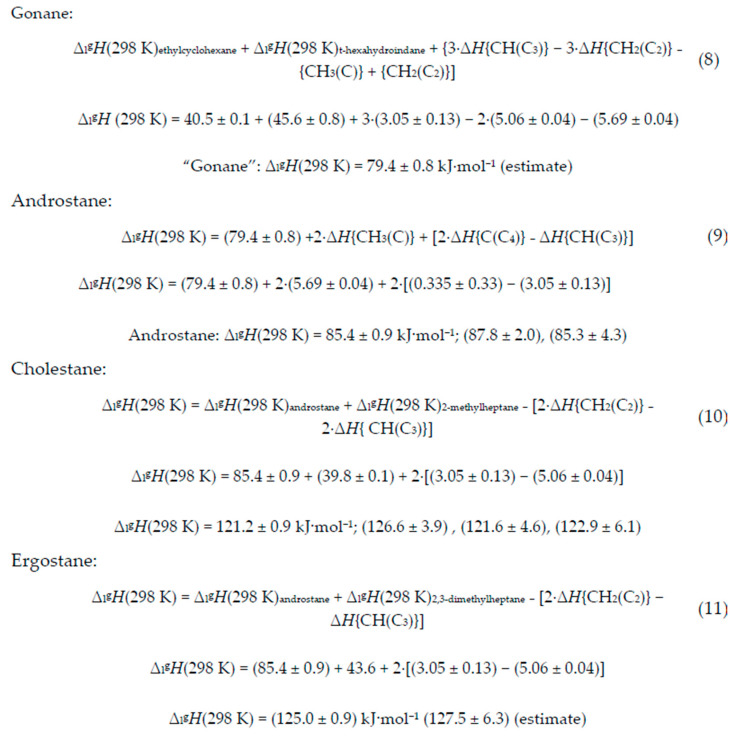
Synthetic analysis of the vaporization enthalpies of “gonane”, “androstane”, “cholestane”, and “ergostane”in kJ.mol^−1^ [16,24,28,29,30,31].

A more complex calculation, the synthetic analysis of “cholestane” starting with two simple synthons, ethylcyclohexane (Δ_l_^g^*H*(298 K) = 40.5 ± 0.1 kJ.mol^−1^ [28]) and *trans*-hexahydroindane (Δ_l_^g^*H*(360 K) = 41.6 kJ.mol^−1^ [29]), is illustrated in Figure 3. The first step of the synthetic analysis combines ethylcyclohexane with *trans*-hexahydroindane to produce “gonane”, the parent hydrocarbon of the steroid nucleus (Δ_l_^g^*H*(298 K) = 79.4 ± 0.8 kJ.mol^−1^). In the process, three cyclic tertiary centers and a cyclic secondary center are formed at the expense of a loss of three cyclic secondary centers and a primary center. No experimental data are currently available for “gonane”. Quotation marks are used in these analyses since no stereochemistry is implied unless specified in the structure of the starting materials. Scheme 3, Equation (8), summarizes the calculations involved in the synthetic analysis of gonane. The second step in the synthetic scheme, summarized by Equation (9), involving the formation of androstane, includes the addition of two methyl groups and the formation of two quaternary centers at the expense of two tertiary centers. A value of 85.4 ± 0.9 kJ.mol^−1^ is evaluated which compares to 87.8 ± 2.0 [30], which was evaluated recently.

The last step of the transformation, described via Equation (10), includes the addition of 2-methylheptane (Δ_l_^g^*H*(298 K) = 39.8 ± 0.1). The addition occurs at positions 6 of the alkane and 17 of the steroid nucleus. This process produces two tertiary carbons at the expense of two methylene groups. The estimated vaporization enthalpy of “cholestane” of 121.1 ± 0.9 kJ.mol^−1^ compares to values of 126.6 ± 3.9 [30] and 121.6 ± 4.6 kJ.mol^−1^ [30] using data from [31] for 5α-cholestane. If, in the last step, 2,3-dimethylheptane (Δ_l_^g^*H*(298 K) = 43.6 ± 0.1 kJ.mol^−1^ [26]), is substituted for 2-methylheptane, a vaporization enthalpy of 125.0 ± 0.9 kJ.mol^−1^ is predicted for the vaporization enthalpy of “ergostane” via synthetic analysis (Figure 3). A value of 129.5 ± 0.9 kJ.mol^−1^ is estimated for “stigmastane” (Scheme S3, pg. S10, S11 (SM)). We are not aware of any experimental data reported previously for either “ergostane” or “stigmastane”.

## 3. Evaluation of Δ_l_^g^*H*(298 K) of PMC at *T* = 298.15 K via Correlation Gas Chromatography

Having illustrated some examples of the manner in which synthetic analysis can provide an alternative method of estimating vaporization enthalpies of larger systems, we now address the issue of whether n-alkanes can be used as standards to evaluate vaporization enthalpies of substituted hydrocarbons. As discussed above, the idea is to use a surrogate whose vaporization enthalpy can or has been evaluated previously. PMC is such a substance having been studied by Bernades et al. [2]. Several of the thermodynamic properties evaluated by these authors are listed on the left side of Table 3, while properties either estimated or derived from the available data are listed on the right side of the table. The vaporization enthalpy of PMC at *T* = 298.15 was derived in two ways: first, by adjusting the fusion enthalpy to *T* = 298.15 K using Equation (12) [14]. This resulted in a fusion enthalpy of 22 ± 1.0 kJ·mol^−1^ (Scheme S2A, SM). An uncertainty of 20% in the temperature adjustment was assumed. Subtracting this value from the sublimation enthalpy also at *T* = 298.15 K using Equation (13) resulted in a value of Δ_l_^g^*H*(298.15 K) = 85.4 ± 1.3 kJ·mol^−1^ (Scheme S2B, SM).

Δ_cr_^l^*H*(298.15 K) = Δ_cr_^l^*H*(*T*_fus_) + [0.15.C*_p_*_(cr)_(298.15 K) − 0.26.*C_p_*_(l)_(298.15 K) –9.83].(*T*_fus_/K − 298.15 K)/1000 (12)

Δ_l_^g^*H*(298.15 K) = Δ_cr_^g^*H*(298.15 K) − Δ_cr_^l^*H*(298.15 K) (13)

Δ_cr_^g^*H*(*T*_2_/K) = Δ_cr_^g^*H*(*T*_1_/K) + (0.75 + 0.15.C*_p_*_(cr)_(298.15 K)).(*T*_2_ − *T*_1_)/1000 (14)

Δ_l_^g^*H*(*T*_2_/K) = Δ_l_^g^*H*(*T*_1_/K) + (10.58 + 0.26.C*_p_*_(l)_(298.15 K)).(*T*_2_ − *T*_1_)/1000 (15)

As an alternate method of evaluating Δ_l_^g^*H*(298.15 K) of PMC, the average sublimation enthalpy evaluated at *T* = 341.5 K of 105.9 ± 1.3 kJ.mol^−1^ [2] was first adjusted to *T*_fus_, also treated as the triple point temperature, using Equation (14) [14]. This resulted in a sublimation enthalpy of 104.8 ± 1.3 kJ·mol^−1^ (Scheme S2C, SM). Subtraction of the fusion enthalpy at *T*_fus_ using an equation analogous to Equation (13) provided a vaporization enthalpy of 77.8 ± 1.3 kJ·mol^−1^ at *T*_fus_ (Scheme S2D, SM). Adjusting the vaporization enthalpy to *T* = 298.15 K using Equation (15) [14] and an estimated liquid heat capacity of 429.1 kJ.mol^−1^.K^−1^ (Scheme S2E, SM) resulted in a vaporization enthalpy of 86.0 ± 1.6 kJ.mol^−1^ (Scheme S2F, SI). Averaging the two values results in a value of 85.8 ± 1.5 kJ·mol^−1^ at *T* = 298.15 K for the vaporization enthalpy of PMC (Scheme S2F, SM). Table 4 summarizes the results of the estimations. The sum of the fusion and vaporization enthalpies in the table are in agreement within the uncertainty in the sublimation enthalpy. The group values used in estimating the heat capacity of liquid PMC are provided in Tables S2 and S3 of the SI.

## 4. Experimental Methods

Table 5 identifies all the materials used in this study as well as their source and analysis. PMC, a solid, purchased from Sigma Aldrich (St. Louis, MO, USA), was available at a mass fraction of 0.97. An analytical sample of Δ^9^-THC in methanol (1 mg/mL) and (±) α-TOC were purchased from Supelco/Aldrich (St. Louis, MO, USA) at mass fractions of 0.90 and 0.96, respectively. The purity of Δ^9^-THC was evaluated using gas chromatography. As noted above, a maximum of 8 stereoisomers are present in (±) α-tocopherol, 4 of which are diastereomers. Figures S1 and S2 (pp. S13–S14) in the Supporting Information illustrate typical gas chromatograms obtained for the targets. A single well-resolved composite peak for (±) α-TOC was obtained at the temperatures studied.

### 4.1. Methods

All experiments were conducted on an HP 5890 Series II gas chromatograph on a 12 m HP-1 column using helium as the carrier gas at a split ratio of approximately 80/1. Temperature was controlled by the instrument to ±0.1 K as monitored using a wide range temperature probe connected to a Go Link! Interface. Residence times of the analytes, *t*_r_, were determined via the difference between each of their respective retention times and the retention time of the methylene chloride which was not retained by the column at the temperatures of the experiments. Details including experimental retention times, slopes, and intercepts from plots of ln(*t*_o_/*t*_r_) vs. K/*T* where *t*_o_ = 60 s and the associated uncertainties are included in the SM for all experiments discussed below (Tables S4A,B–S11A,B).

### 4.2. Evaluation of Vaporization Enthalpy

Values of ln(*t*_o_/*t*_r_) were plotted as a function of K/*T* over a 30 K range at 5 K intervals. In addition to the methylene chloride, some methanol was also present when using Δ^9^-THC. All plots resulted in linear relationships characterized by coefficients of determination, r^2^ > 0.9998. Enthalpies of transfer (Δ_trn_*H*(*T*_m_)) were calculated as the product of the absolute value of the slope of the line and the gas constant (*R* = 8.314 J·K^−1^·mol^−1^). Enthalpies of transfer are related to the vaporization enthalpy (Δ_l_^g^*H*(*T*_m_)) via Equation (16) where Δ_intr_*H*(*T*_m_) refers to the enthalpy of interaction of the solute with the solid support. Plots of Δ_l_^g^*H*(298.15 K) versus Δ_trn_*H*(*T*_m_) of the standards resulted in linear relationships which were used to evaluate Δ_l_^g^*H*(298.15 K) of the targets. All runs were performed in duplicate. Correlations are provided in the SM as Tables S4B–S11B. PMC was treated as a target in all runs when it was included in the mix. The vaporization enthalpies of the standards are reported in Table 6 below, together with the constants used to evaluate their vapor pressures.

Δ_trn_*H*(*T*_m_) = Δ_l_^g^*H*(*T*_m_) + Δ_intr_*H*(*T*_m_) (16)

### 4.3. Evaluation of Vapor Pressure

Vapor pressures can also be evaluated for hydrocarbons when n-alkanes are used as standards. In this instance, as a consequence of the alkane adjustment, there is no basis to assume that the vapor pressures normally evaluated using n-alkanes as standards would produce relevant values for the compounds of this study. However, as discussed below, given the fact that the n-alkane adjustment was found to be relatively small, vapor pressures were also evaluated for all three compounds, in particular for comparison to available experimental and estimated values for Δ^9^-THC, experimental data for the tocopherols, and at the fusion temperature for PMC. The vapor pressure constants of the Cox Equation, Equation (17) [20], and those of a third order polynomial, Equation (18) [21,22], used for evaluating vapor pressures of the n-alkanes used as standards, are provided in Table 6. Also included are the constants of Equation (19) [32] used for evaluating liquid vapor pressures of the tocopherols as a class of compounds and Equation (20) for evaluating the vapor pressure of PMC at *T*_fus_; this vapor pressure is reported in Table 3. Unlike α-TOC which is a mixture of diastereomers, vapor pressures and vaporization enthalpies of the tocopherols (a mixture of α, β, γ, and δ tocopherol) also vary in the number of methyl groups on the aromatic ring, 3, 2, 2, 1, respectively, and are included as an approximate value for comparison [32].

ln(*p*/*p*^o^) = (1 − *T*_o_/*T*).exp(A_o_ + A_1_.*T*^−1^ + A_2_.*T*^−2^); *p*^o^ = 101,325 Pa (17)

ln(*p*/*p*^o^) = A.*T*^−3^ + B.*T*^−2^ + C.*T*^−1^ + D (18)

ln(*p*/Pa) = A_3_ + B_3_/(*T*) + C_3_.ln(*T*) + D_3_.*T* ^E3^; (19)

ln(*p*/Pa) = a + b/*T* (20)

### 4.4. Uncertainties

All uncertainties refer to one standard deviation and are equivalent to the standard uncertainties as defined by the Guide to the Expression of Uncertainty in Measurement [34]. All slopes and intercepts were calculated via linear regression. Uncertainties of all combined results were calculated as (*u*_1_^2^ + *u*_2_^2^ + …)^0.5^. Uncertainties reported for correlations are only a measure of the quality of the correlation. Uncertainties reported for values evaluated from logarithmic terms are reported as an average value of the two uncertainties evaluated.

## 5. Experimental Results

### 5.1. Vaporization Enthalpies of PMC, Δ^9^-THC) and α-TOC at T = 298.15 via Correlation Gas Chromatography

Table 7 illustrates the results of three different correlations using a series of n-alkanes from n-hexadecane to n-dotriacontane to evaluate the vaporization enthalpies of PMC, Δ^9^-THC, and α-TOC. Duplicate runs are provided in the Supporting Information. Equations (21)–(23) quantify the quality of the correlations. A summary of the results of all four sets of correlations for PMC, Δ^9^-THC, and α-TOC is provided in Table 8.

Comparing the vaporization enthalpy of PMC of 85.8 ± 1.5 kJ·mol^−1^ as derived from the work of Bernades et al. [2] to the average value of 83.3 ± 1.1 kJ·mol^−1^ obtained in this work, the use of n-alkanes as standards appear to underestimate the value of PMC by 2.5 ± 1.8 kJ·mol^−1^. Adjusting both Δ^9^-THC and α-TOC by this amount suggests that their “alkane-adjusted” vaporization enthalpies to be 120.4 ± 1.8 kJ·mol^−1^ for Δ^9^-THC and 150.7 ± 2.6 kJ·mol^−1^ for α-TOC. It should be emphasized that the vaporization enthalpy for α-TOC is an ensemble average of all diastereomers present.

### 5.2. Vapor Pressures of PMC, Δ^9^-THC, and α-TOC at T = 298.15 via Correlation Gas Chromatography Using n-Alkanes as Standards

Values of (*t*_o_/*t*_r_)_avg_ of both standards and targets evaluated from the slopes and intercepts of duplicate runs were averaged. Vapor pressures were evaluated by correlating values of ln(*t*_o_/*t*_r_)_avg_ of the standards against their corresponding vapor pressures in the form of ln(*p*/*p*^o^), where *p*^o^ refers to the reference pressure, 101,325 Pa. The values of ln(*p*/*p*^o^) of the targets were evaluated from the resulting slopes and intercepts and their respective value of (*t*_o_/*t*_r_)_avg_. The results of correlating values of ln(*t*_o_/*t*_a_)_avg_ of the standards against their corresponding vapor pressures for all three targets at *T* = 298.15 K are illustrated in Table 9A–C. Equations (24)–(26) summarize the quality of the correlations. Similar correlations were performed at *T* = 310 K and at 10 K increments up to 400 K. Correlation coefficients for all correlations (r^2^) exceeded 0.9998. Since PMC was used as the surrogate, it was also included as a target in each run analyzed. All vapor pressures of the targets evaluated over the temperature range (298.15 to 400) K were then fit to a second order polynomial, Equation (27). The resulting constants are reported in Table 10. Also included in Table 10 are the constants evaluated from an average of the six runs that included PMC as a target.

ln(*p*/*p*^o^) = A + B/(*T*/K) + C/(*T*/K)^2^ (27)

## 6. Vaporization Enthalpies of α-TOC and Δ^9^-THC via Synthetic and Retrosynthetic Analysis

### 6.1. Estimation of α-TOC via Synthetic Analysis

**Figure 4:**
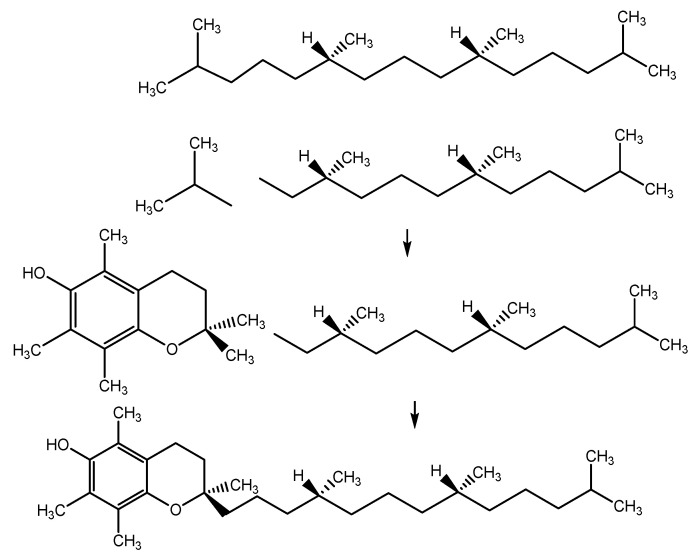
A synthetic analysis scheme for validating the vaporization enthalpy results from correlation gas chromatography for “α-TOC”. No stereochemistry implied.

**Scheme 4:**
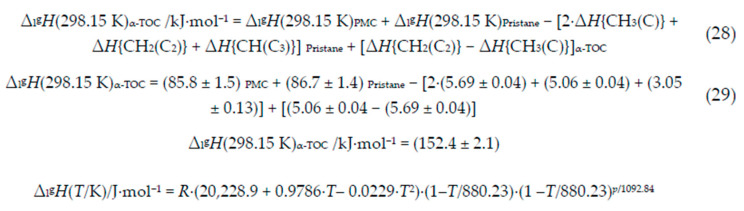
Conversion of PMC and pristane to “α-TOC”.

Figure 4 illustrates a synthetic analytic protocol for evaluating the vaporization enthalpy of “α-TOC” similar to the one used for the conversion of “androstane” to “cholestane”. Pristane, an interesting C_19_H_40_ hydrocarbon, is reported to have a vaporization enthalpy of 86.7 ± 1.4 kJ·mol^−1^ at *T* = 298.15 K [36]. Synthetic analysis of “α-TOC” using pristane requires the loss of an isobutyl group, calculated as the loss of [2·Δ*H*{CH_3_(C)}, Δ*H*{CH(C_3_)}, and Δ*H*{CH_2_(C_2_)}], and from PMC, the conversion of a Δ*H*{CH_3_(C)} group to a Δ*H*{CH_2_(C_2_)}, Equation (28). The calculation is summarized in Scheme 4. The vaporization enthalpy of “α-TOC” evaluated via correlation gas chromatography adjusted for the “alkane increment” is 150.7 ± 2.7 kJ·mol^−1^ compares to an estimated value of 152.4 ± 2.1 kJ·mol^−1^; both are in good agreement. An approximate method for evaluating the vaporization enthalpy of TOCs as a function of temperature has been reported by Damaceno et al. [32] using Equation (29). The results of using this equation are discussed below.

### 6.2. Estimation of the Vaporization Enthalpy of Δ^9^-THC via Synthetic Analysis

**Figure 5:**
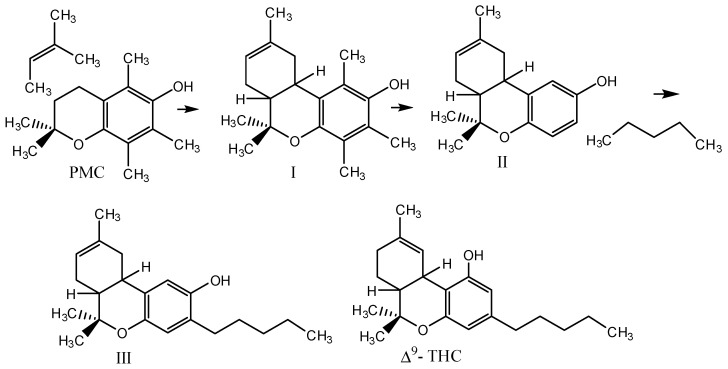
Combination of a synthetic and retrosynthetic analysis scheme for the analysis of the vaporization enthalpy of Δ^9^-tetrahydrocannabinol. No stereochemistry is implied.

**Scheme 5:**
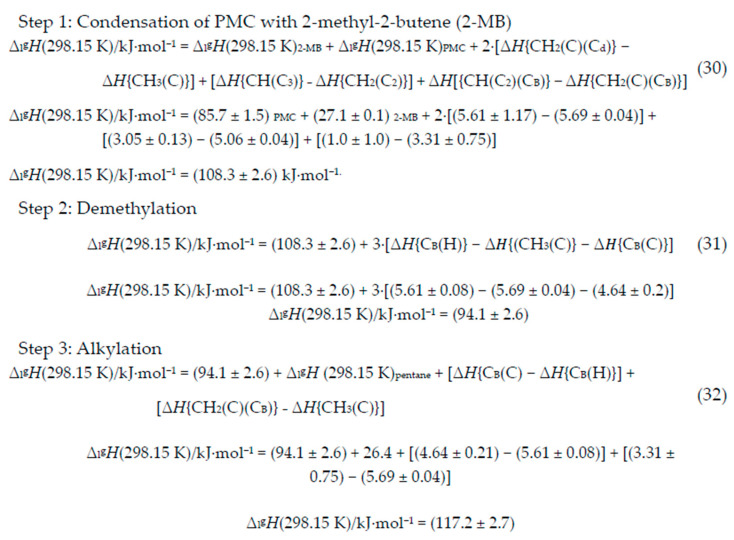
Conversion of PMC to “Δ^9^-THC” [20,26].

Estimation of the vaporization enthalpy of Δ^9^-THC from PMC is somewhat more problematic. A possible pathway is provided in Figure 5 and described in Scheme 5. Step 1 involves the formation of adduct I and follows a protocol similar to the one reported for the evaluation of gonane, Equation (30). A vaporization enthalpy of (108.3 ± 2.6) kJ·mol^−1^ is obtained.

Adduct II involves complete removal of the aromatic methyl groups, as seen in Equation (32). A value of 94.2 ± 2.6 kJ·mol^−1^ results. Adduct III inserts the five carbons of pentane appropriately modified ortho to the phenolic oxygen and meta to the ether linkage, as seen in Equation (32). At this point, synthetic adduct III is isomeric with Δ^9^-THC. The value of 117.2 ± 2.7 kJ·mol^−1^ compares quite well with the value of 120.3 ± 1.8 kJ·mol^−1^ evaluated using correlation gas chromatography for Δ^9^-THC adjusted for the “alkane increment”.

An alternative pathway using 2-methyl-1-butene while providing a closer related structural isomer is precluded by the unavailability of a necessary group value. Isomerization of the double bond in Figure 5 is also precluded by the absence of a value for the same group, CH(C)(C_B_)(C_d_). Assuming double bond isomerization contributes very little change to the vaporization enthalpy, and unless there is a significant change in hydrogen bonding, a shift in the position of the phenolic OH is also not likely to produce a significant change. Granting these two assumptions to be reasonable, the estimated vaporization enthalpy of Δ^9^-THC of 117.2 ± 2.7 kJ·mol^−1^ is within the experimental uncertainties of the value of 120.4 ± 1.3 kJ·mol^−1^ evaluated via correlation using the alkane adjustment. An alternative pathway results in a similar but slightly different value. For example, removal of only two of the methyl groups, at positions 5 and 8 of the aromatic ring in step 2, and condensation with butane, results in a value of 118.3 ± 2.8 kJ·mol^−1^. This estimation is provided in Scheme S4 (SM). Isomerization of the phenolic hydroxide to position 5 of the aromatic ring provides an estimate of the vaporization enthalpy of Δ^8^-THC.

## 7. Liquid Vapor Pressures of PMC, Δ^9^-THC, and α-TOC

Experimental liquid vapor pressures of the targets are available for Δ^9^-THC, for α-tocopherols, as a boiling temperature at 1.1 kPa for α-TOC, and at the triple point for PMC. As reported in Table 3, extrapolation of the experimental vapor pressure of crystalline PMC to *T*_fus_, also treated as the triple-point temperature, resulted in a vapor pressure of 12.2 Pa at *T* = 365.3. The vapor pressure evaluated via the constants of Equation (27), as reported in Table 10 for PMC at this temperature for runs (S1–S6), is 22 Pa.

**Figure 6:**
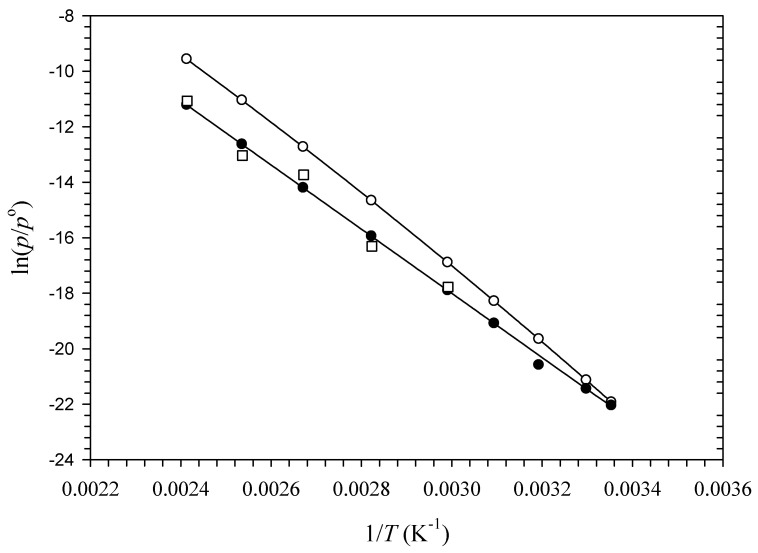
A plot of ln(*p*/*p^o^*) versus K/*T* for Δ^9^-THC.; circles (ο): this work; solid circles (●): estimated values; squares (□): experimental data [9]; (1/298.15 = 0.00335).

Both experimental (□) and estimated vapor pressures (●) for Δ^9^-THC have been reported covering a temperature range from 298.15 to 414 K [9]. Figure 6 provides a comparison of both to those evaluated in this work (o) with Equation (27). Agreement at *T* = 298.15 K with the estimated value reported by Lovestead and Bruno [9] with that evaluated in this work, (Equation (27), line and circle) is quite good 3.0·10^−5^ Pa [this work], versus 2.6·10^−5^ Pa [9] (Table 10). Values diverge as the temperature increases. Since the experimental and estimated values reported by reference [9] correlate quite well with each other, we have used both sets of data to estimate the vaporization enthalpy at the mean temperature reported: Δ_l_^g^*H*(356.2 K)/kJ·mol^−1^ = 96.9 ± 1.7 kJ·mol^−1^ (r^2^ = 0.9963). Adjusted to *T* = 298.15 K using Equation (15) and an estimated liquid heat capacity of 610.1 J·mol^−1^·K^−1^ [Scheme S5, SM] for Δ^9^-THC results in Δ_l_^g^*H*(298.15 K) = 106.7 ± 2.6 kJ·mol^−1^ using the data from [9]. Using only experimental data from [9] results in a value of only 95.9 ± 14.5 kJ·mol^−1^. A similar set of calculations using the constants of Equation (27) for Δ^9^-THC evaluated in this work results in a vaporization enthalpy of Δ_l_^g^*H* (356.2 K) = 109.0 ± 0.8 kJ·mol^−1^ at *T* = 356.2 K and 118.8 ± 2.1 kJ·mol^−1^ at *T* = 298.15 K. An uncertainty of 20% in the temperature adjustment for both sets of calculations is assumed. Comparison of the change in vaporization enthalpy going from PMC (85.7 ± 1.4) to Δ^9^-THC (106.7 ± 2.6) kJ·mol^−1^ results in a change of 21 kJ·mol^−1^ for a C_7_H_10_ increment in molecular formula using values from Lovestead and Bruno [9]; a change of 33 kJ·mol^−1^ is calculated using the values evaluated in this work using Equation (27). Experimental vaporization enthalpies of C_7_H_10_ hydrocarbons at *T* = 298.15 K fall in the 30+ kJ·mol^−1^ range [14]. If this C_7_H_10_ increment contributes equally to the vaporization enthalpy as suggested by the synthetic analysis estimations posted above, a value of about = 106.7 kJ·mol^−1^ for Δ^9^-THC seems somewhat low.

**Figure 7:**
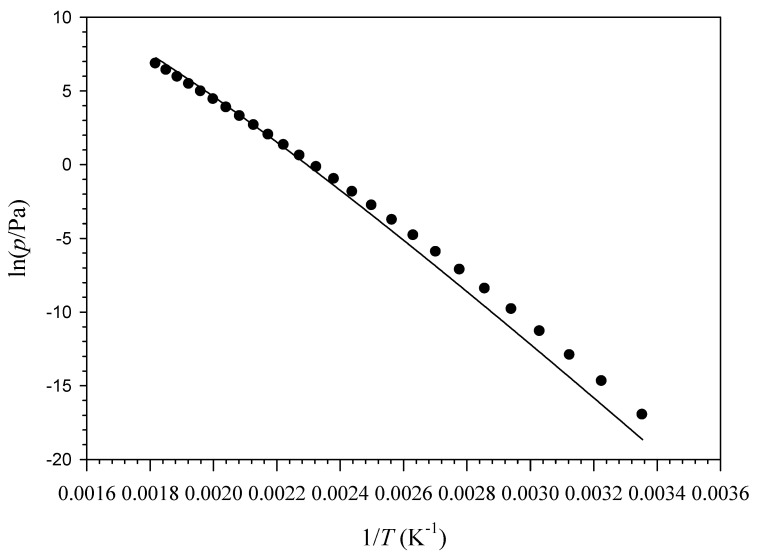
(**^__^**): Vapor pressure for the tocopherols as a group calculated using Equation (19) [32]; ●: vapor pressures evaluated for α-TOC using Equation (27) (this work) and the constants in Table 10. A vapor pressure comparison is available in the SM (p. S12).

Vapor pressures of the tocopherols have been reported as a group using Equation (19) and also individually as a boiling temperature at a pressure of 1.1 kPa. For α-tocopherol, a boiling temperature of *T* = 549.3 K has been reported at this pressure [32]. Using Equation (27) and the constants reported in Table 10 for α-tocopherol, a vapor pressure of 1.1 kPa is calculated at *T* = 554 K, in reasonable agreement with the literature value. Despite differences in the number of methyl groups present on the aromatic ring, the maximum boiling point difference reported between the four tocopherols was less than 5 K at this pressure [32]. A comparison of the vapor pressures evaluated with Equation (19) and this work, as in Equation (27), is provided in Figure 7. At *T* = 298.15 K, a vapor pressures of 8·10^−9^ Pa [32] calculated with Equation (19) for the tocopherols compares to a value of 4·10^−8^ Pa evaluated for α-tocopherol by this work.

In addition, using a calculated vapor pressure at *T* = 298.15 K of less than 0.01 kPa in the exponent of Equation (29), a limiting vaporization enthalpy of 153 kJ·mol^−1^ is calculated for the tocopherols as a group. This compares with the vaporization enthalpy values of (152.8 ± 2.3 and 150.8 ± 2.8) kJ·mol^−1^ evaluated for α-tocopherol via synthetic analysis and correlation gas chromatography, respectively.

## 8. Summary

This article proposes a synthetic method based on group additivity combined with experimental measurements to evaluate the vaporization enthalpies of two moderately large molecules that could be eluted through a gas chromatographic column using a series of hydrocarbon standards and a surrogate with known properties, provided certain requirements as described above are satisfied. The proposed method is applied to racemic α-tocopherol which is a mixture of several diastereomers and a positional isomer of Δ^9^-THC. While all numbers evaluated are approximate, given their magnitude, they are unlikely to differ by more than a few percent from their present values as a result of the isomeric components present. In this instance, the vaporization enthalpy evaluated with n-alkanes for the surrogate is quite similar to its value in the literature. This similarity is also reflected in the resultant vapor pressures of the target substances that appear comparable to available experimental values. How well using the “alkane increment” method proposed responds to instances when significant differences in vaporization enthalpy is obtained between the surrogate and the n-alkanes remains to be determined. The use of synthetic and retrosynthetic analysis does appear to provide a simple method of estimating reliable vaporization enthalpies of substances using experimental data of smaller components. The method proposed has also been used to estimate vaporization enthalpies of two steroidal hydrocarbons that have not yet been studied. It could also prove useful for evaluating similar properties of other substances exhibiting volatilities too low to be evaluated by current conventional methods. In conclusion, mean vaporization enthalpies of (118.6 ± 2.4) kJ·mol^−1^ (3 entries) and (151.8 ± 2.5) kJ·mol^−1^ (2 entries) are estimated for Δ^9^-THC and α-TOC, respectively.

## Figures and Tables

**Table 1:** Group values and their uncertainties from Guthrie and Taylor [18] (kJ·mol^−1^).

	Group Value	Uncertainty	Group	Group Value	Uncertainty
CH_3_(C)	5.69	±0.04	C_d_H_2_	4.18	±1.5
CH_2_(C_2_)	5.06	±0.04	C_d_H(C)	5.15	±1.13
CH(C_3_)	3.05	±0.13	C_d_(C_2_)	4.85	±1.17
C(C_4_)	0.335	±0.33	C_B_(H)	5.61	±0.08
CH_2_(C)(C_d_)	5.61	±1.17	C_B_(C)	4.64	±0.21
CH(C_2_)(C_d_)	4.77	±4.39	CH_2_(C)(C_B_)	3.31	±0.75
C(C_3_)(C_d_)	−1.21	±2.97	CH(C_2_)(C_B_)	1.00	±1.00

**Table 2:** A comparison of the vaporization enthalpies of n-alkanes, *n*_c_ = C_10_ to C_40_, evaluated via synthetic analysis with experimental values in kJ·mol^−1^.

n-Alkane	Δ_l_^g^*H*(298.15 K) Calcd. Lit. ^a^		n-Alkane	Δ_l_^g^*H*(298.15 K) Calcd. Lit. ^a^	
n-Pentane		26.42 ± 0.14	n-Tricosane	116.8 ± 0.8	117.0 ± 2.8 ^b^
n-Hexane		31.52 ± 0.16	n-Tetracosane	121.8 ± 0.9	121.9 ± 2.8 ^b^
n-Heptane		36.57 ± 0.18	n-Pentacosane	126.9 ± 0.9	126.8 ± 2.9 ^b^
n-Octane		41.56 ± 0.21	n-Hexacosane	132.1 ± 1.0	131.7 ± 3.2 ^b^
n-Nonane		46.55 ± 0.23	n-Heptacosane	137.2 ± 1.0	135.6 ± 3.3 ^b^
n-Decane	51.6 ± 0.5	51.42 ± 0.26	n-Octacosane	142.2 ± 1.1	141.9 ± 4.9 ^b^
n-Undecane	56.7 ± 0.6	56.58 ± 0.57	n-Nonacosane	147.2 ± 1.1	147.1 ± 5.1 ^b^
n-Dodecane	61.8 ± 0.6	61.52 ± 0.62	n-Tricontane	152.3 ± 1.1	152.3 ± 5.3 ^b^
n-Tridecane	66.8 ± 0.7	66.68 ± 0.67	n-Henetriacontane	156.9 ± 1.9	157.2 ± 1.4 ^c^
n-Tetradecane	71.9 ± 0.7	71.73 ± 0.77	n-Dotriacontane	161.4 ± 2.4	162.5 ± 1.4 ^c^
n-Pentadecane	76.9 ± 0.8	76.77 ± 0.81	n-Triatricontane	166.6 ± 2.4	167.6 ± 1.4 ^c^
n-Hexadecane	81.9 ± 0.8	81.35 ± 0.87	n-Tetratriacontane	171.7 ± 2.5	172.7 ± 1.5 ^c^
n-Heptadecane	86.9 ± 0.3	86.47 ± 0.9	n-Pentatriacontane	176.7 ± 2.6	178.1 ± 5.4 ^c^
n-Octadecane	91.8 ± 0.3	91.44 ± 0.9	n-Hexatriaconane	181.6 ± 2.7	182.8 ± 5.5 ^c^
n-Nonadecane	96.7 ± 0.4	96.44 ± 1.0	n-Heptatriacontane	186.6 ± 2.7	187.5 ± 5.6 ^c^
n-Eicosane	101.6 ± 0.5	101.81 ± 1.0	n-Octatriacontane	191.6 ± 2.7	192.6 ± 5.7 ^c^
n-Heneicosane	106.7 ± 0.6	106.8 ± 2.5 ^b^	n-Nonatriacontane	197.0 ± 2.8	NA ^d^
n-Docosane	111.9 ± 0.8	111.9 ± 2.7 ^b^	n-Tetracontane	202.4 ± 2.8	203.5 ± 2.9 ^e^

^a^ Uncertainties represent probable error; ref. [20]. ^b^ Ref. [21]; uncertainties represent 1 standard deviation. ^c^ Ref. [22]; uncertainties represent 1 standard deviation. ^d^ Not available. ^e^ Ref. [23]; uncertainties represent 1 standard deviation.

**Table 3:** A summary of the literature data for PMC [2] and the derived thermodynamic data.

Literature Values [2]		Estimated/Derived Values	
*C*_p,m_(cr)(298.15 K)	315.3 ± 9.6 J.mol^−1^.K^−1^	*C*_p,m_(l)(298.15 K)	429.1 kJ.mol^−1^.K^−1 a^
*T*_fus_ =	365.3 K	Δ_cr_^l^*H*(298.15 K)	22.0 ± 1.0 kJ·mol^−1^
Δ_cr_^l^*H*(*T*_fus_ K)	27.0 ± 0.2 kJ.mol^−1^	Δ_l_^g^*H*(*T*_fus_/K)	77.8 ± 0.9 kJ·mol^−1^
Δ_cr_^g^*H*(341.5 K)	105.9 ± 1.3 kJ.mol^−1 b^	Δ_l_^g^*H*(298.15 K)	85.8 ± 1.5 kJ·mol^−1 b^
Δ_cr_^g^*H*(298.15 K)	107.4 ± 0.8 kJ.mol^−1^	*p_tp_*_=_	12.2 Pa ^c^

^a^ Estimated using the group values reported in Tables S1 and S2, SM [17]; Scheme S2E. ^b^ Average of two independent evaluations. ^c^ Vapor pressure at the melting point treated as the triple-point temperature [2].

**Table 4:** The sublimation enthalpy and estimated vaporization and fusion enthalpies of PMC at *T* = 298.15 K.

Δ_cr_^g^*H*(298.15 K)/kJ·mol^−1^	Δ_cr_^l^*H*(298.15 K)/kJ·mol^−1^	Δ_l_^g^*H*(298.15 K)/kJ·mol^−1^
107.4 ± 0.8 [2]	22.0 ± 1.0 ^a^	85.8 ± 1.5 ^b^

^a^ Scheme S2A, SM. ^b^ Scheme S2B,F, SM.

**Table 5:** Origin and analysis (mass fraction) of the standards and THC.

Compound	CASRN’s	Supplier	Mass Fraction
n-Hexadecane	544-76-3	Sigma/Aldrich	0.99
n-Heptadecane	629-78-7	Sigma/Aldrich	0.99
n-Nonadecane	629-92-5	Sigma/Aldrich	0.99
n-Eicosane	112-95-8	Sigma/Aldrich	0.99
n-Heneicosane	629-94-7	Sigma/Aldrich	0.98
n-Docosane	629-97-0	Sigma/Aldrich	0.99
n-Tetracosane	646-31-1	Sigma/Aldrich	0.99
n-Pentacosane	629-99-2	Sigma/Aldrich	0.99
n-Hexacosane	630-01-3	Sigma/Aldrich	0.99
n-Octacosane	630-02-4	Sigma/Aldrich	0.99
n-Triacontane	638-68-6	Sigma/Aldrich	0.98
n-Dotriacontane	544-85-4	Sigma/Aldrich	0.97
2,2,5,7,8-Pentamethylchroman-6-ol	950-99-2	Sigma/Aldrich	0.97
(-)-*trans* Δ^9^-Tetrahydrocannabinol	1972-08-3	Supelco/Aldrich	0.90 ^a^
(±) α-Tocopherol	10,191-41-0	Supelco/Aldrich	0.96

^a^ Evaluated with gas chromatography.

**Table 6:** Vaporization enthalpies and constants for Equations (17)–(20); *p*^o^ = 101,325 Pa.

Compound	Δ_l_^g^*H*(298.15 K) ^a^ (kJ·mol^−1^)	A_o_	10^3^*·*A_1_(K^−1^)	10^6^·A_2_(K^−2^)		*T*_o_/K	
n-Hexadecane	81.35 ± 0.81	3.18271	−2.002545	1.384476		559.978	
n-Heptadecane	86.47 ± 1.7	3.21826	−2.036553	1.383899		575.375	
n-Nonadecane	96.44 ± 1.9	3.27626	−2.062714	1.346737		603.989	
n-Eicosane ^a^	101.81 ± 2.0	3.31181	−2.102218	1.348780		617.415	
	Δ_l_^g^*H*(298.15 K) ^b^	10^−6^·A(K)^3^	10^−4^·B(K)^2^	C(K)		D	
n-Heneicosane	106.8 ± 2.4	199.89	−290.75	−98.135		6.6591	
n-Docosane	111.9 ± 2.7	217.13	−311.76	110.72		6.5353	
n-Tricosane	117.0 ± 2.8	233.86	−332.2	310.77		6.4198	
n-Tetracosane	121.9 ± 2.8	250.72	−352.86	530.15		6.2817	
n-Pentacosane	126.8 ± 2.9	267.38	−373.07	741.19		6.1496	
n-Hexacosane	131.7 ± 3.2	282.44	−391.93	910.53		6.0704	
n-Octacosane	141.9 ± 4.9	313.89	−431.20	1279.4		5.8835	
n-Triacontane	152.3 ± 5.3	334.04	−469.98	1601.6		5.7696	
n-Dotriacontane	162.5 ± 2.8 ^c^	375.24	−509.21	1947.2		5.6303	
Equation (19)	Δ_l_^g^*H*(298.15 K)	A_3_ ^e^	B_3_ ^d,e^	C_3_ ^d,e^	D_3_ ^d,e^		E_3_ ^d,e^
TOC	153.7 ^d^	50.449	−20,228.9	0.9786	− 0.0229		1
Equation (20)	Δ_cr_^g^*H*(298.15 K)	a		b			
PMC ^f^	107.4 ± 0.8	37.35 ± 0.24		(12,728.8 ± 80.6)			

^a^ Reference [20]; uncertainty represents probable error. ^b^ Reference [21]; uncertainty represents probable error. ^c^ Reference [22]. ^d^ Reference [32]; evaluated for the tocopherols, (α, β, γ, and δ) as a class of compounds. ^e^ Reference [33]; evaluated for α-tocopherol. ^f^ Reference [2].

**Table 7:** Evaluation of the Δ_l_^g^*H*(298 K) of PMC, Δ^9^-THC, and α-TOC using n-alkanes ^a^.

Run S1	–Slope *T*/K	Intercept	Δ*H*_trn_(468 K) kJ·mol^−1^	Δ_l_^g^*H*(298 K) kJ·mol^−1^ (lit) ^b^	Δ_l_^g^*H*(298 K) kJ·mol^−1^ (calc)	
n-Hexadecane	6293.4 ± 22	12.933 ± 0.047	52.32 ± 0.18	81.35 ± 0.8	81.4 ± 1.1	
n-Heptadecane	6680.9 ± 22	13.385 ± 0.047	55.54 ± 0.18	86.47 ± 1.7	86.3 ± 1.1	
PMC	6405.4 ± 18	12.489 ± 0.039	53.25 ± 0.15		82.8 ± 1.1	
n-Nonadecane	7484.9 ± 25	14.357 ± 0.054	62.23 ± 0.21	96.44 ± 1.9	96.7 ± 1.2	
n-Eicosane	7876.3 ± 25	14.828 ± 0.053	65.48 ± 0.20	101.81 ± 2.0	101.8 ± 1.2	
n-Heneicosane	8259.4 ± 0.27	15.28 ± 0.057	68.67 ± 0.22	106.8 ± 2.2 ^c^	106.7 ± 1.2	
Δ_l_^g^*H*(298.15 K)/kJ·mol^−1^ = (1.55 ± 0.014)Δ*H*_trn_(468 K) + 0.211 ± 0.0.83) r^2^ = 0.9998						(21)
**Run S5**	**–Slope** ***T*/K**	**Intercept**	**Δ*H*_trn_(503 K)** **kJ·mol^−1^**	**Δ_l_^g^*H*(298 K)** **kJ·mol^−1^ (lit) ^c^**	**Δ_l_^g^*H*(298 K)** **kJ·mol^−1^ (calc)**	
PMC	6102.8 ± 41	11.858 ± 0.081	50.74 ± 0.34		84.1 ± 0.4	
n-Eicosane	7426.9 ± 37	13.898 ± 0.074	61.74 ± 0.31	101.81 ± 2.0 ^b^	101.5 ± 0.4	
n-Heneicosane	7790.2 ± 36	14.314 ± 0.072	64.76 ± 0.30	106.8 ± 2.2	106.4 ± 0.4	
n-Docosane	8178.0 ± 35	14.781 ± 0.071	67.99 ± 0.30	111.9 ± 2.7	111.7 ± 0.4	
n-Tetracosane	8927.0 ± 37	15.667 ± 0.074	74.22 ± 0.31	121.9 ± 2.8	122.0 ± 0.4	
Δ^9^-THC	8620.8 ± 40	14.835 ± 0.080	71.67 ± 0.33		117.8 ± 0.4	
n-Pentacosane	9292.8 ± 38	16.095 ± 0.075	77.26 ± 0.32	126.8 ± 2.9	127.0 ± 0.4	
n-Hexacosane	9649.0 ± 42	16.506 ± 0.83	80.22 ± 0.35	131.7 ± 3.2	131.9 ± 0.5	
Δ_l_^g^*H*(298.15 K)/kJ·mol^−1^ = (1.612 ± 0.004)Δ*H*_trn_(503 K) + 2.306 ± 0.33 r^2^ = 0.9997						(22)
**Run S7**	**–Slope** ***T*/K**	**Intercept**	**Δ*H*_trn_(503 K)** **kJ·mol^−1^**	**Δ_l_^g^*H*(298 K)** **kJ·mol^−1^ (lit) ^c^**	**Δ_l_^g^*H*(298 K)** **kJ·mol^−1^ (calc)**	
PMC	5951.0 ± 28	11.557 ± 0.052	49.47 ± 0.23		82.1 ± 0.5	
n-Tetracosane	8688.8 ± 29	15.191 ± 0.055	72.24 ± 0.24	121.7 ± 2.8	121.7 ± 0.6	
n-Pentacosane	9331.1 ± 30	16.180 ± 0.059	77.58 ± 0.25	126.8 ± 2.9	126.9 ± 1.3	
n-Hexacosane	9704.2 ± 24	16.625 ± 0.047	80.68 ± 0.20	131.7 ± 3.2	132.0 ± 1.3	
n-Octacosane	10,133.2 ± 32	16.875 ± 0.060	84.24 ± 0.27	141.9 ± 4.9	142.2 ± 0.7	
(±) α-Tocopherol	10,566.6 ± 42	16.938 ± 0.078	87.85 ± 0.35		148.3 ± 0.7	
Triacontane	10,845.3 ± 31	17.708 ± 0.058	90.16 ± 0.26	152.3 ± 0.6	152.3 ± 0.7	
n-Dotriacontane	11,557.6 ± 36	18.544 ± 0.068	96.09 ± 0.30	162.5 ± 0.7 ^d^	162.4 ± 0.7	
Δ_l_^g^*H*(298.15 K)/kJ·mol^−1^ = (1.705 ± 0.005)Δ*H*_trn_(533 K) − (1.424 ± 0.456) r^2^ = 0.9999						(23)

^a^ Uncertainties represent one standard deviation unless noted otherwise. ^b^ Reference [20]; uncertainties represent probable error unless noted otherwise. ^c^ Reference [21] unless noted otherwise. ^d^ Reference [22].

**Table 8:** A summary of the vaporization enthalpies evaluated via correlation gas chromatography using n-alkane standards for PMC, Δ^9^-THC, and α-TOC in kJ·mol^−1^ at *T* = 298.15 K (Tables S4B–S11B, SM).

Run	PMC	Δ^9^-THC	α-TOC
S1	82.8 ± 1.1		
S2	82.9 ± 1.1		
S3		117.9 ± 0.3	
S4		118.1 ± 0.4	
S5	84.1 ± 0.4	117.8 ± 0.4	
S6	84.0 ± 0.3	117.6 ± 0.3	
S7	82.7 ± 1.7		148.1 ± 2.0
S8	83.0 ± 1.4		148.3 ± 1.8
Average	83.3 ± 1.1	117.9 ± 0.4	148.2 ± 1.9
“Alkane Adjusted”	85.7 ± 2.2	120.4 ± 1.8	150.7 ± 2.6

**Table 9:** (A–C) Correlations of ln(*p*/*p*^o^) with ln(*t*_o_/*t*_a_)_avg_ of the standards at *T* = 298.15 K; evaluation of the liquid vapor pressures of the targets; uncertainties represent one standard deviation.

9A	ln(*t*_o_/*t*_a_)_avg_	ln(*p*/*p*^o^)	ln(*p*/*p*^o^)_calc_	10^4^·*p*/Pa	10^4^·*p*/Pa(lit) ^a^	
n-Hexadecane	−8.150	−13.181	−13.18 ± 0.10	1920 ± 190	1910	
n-Heptadecane	−9.002	−14.315	−14.30 ± 0.10	620 ± 64	615	
PMC	−8.977		−14.27 ± 0.10	640 ± 66		
n-Nonadecane	−10.737	−16.551	−16.59 ± 0.11	63 ± 6.8	66	
n-Eicosane	−11.579	−17.696	−17.70 ± 0.12	21 ± 2.4	21	
n-Heneicosane	−12.425	−18.836	−18.81 ± 0.12	6.8 ± 0.82	6.7	
ln(*p*/*p*^o^) = (1.318 ± 0.007) ln(*t*_o_/*t*_a_)_avg_ − (2.43 ± 0.08) r^2^ = 0.9999						(24)
**9B**				**10^4^·*p*/Pa**	**10^4^·*p*/Pa**	
PMC	−8.620		−14.45 ± 0.08	530		
n-Eicosane	−11.027	−17.696	−17.72 ± 0.09	20	21	
n-Heneicosane	−11.839	−18.836	−18.83 ± 0.09	6.8	6.7 ^b^	
n-Docosane	−12.668	−19.972	−19.95 ± 0.10	2.2	2.1 ^b^	
n-Tetracosane	−14.291	−22.175	−22.16 ± 0.10	0.24	0.24 ^b^	
Δ^9^-THC	−14.089		−21.88 ± 0.10	0.32	0.26 ^c^	
n-Pentacosane	−15.095	−23.244	−23.25 ± 0.10	0.08	0.08 ^b^	
n-Hexacosane	−15.890	−24.309	−24.33 ± 0.11	0.028	0.028 ^b^	
ln(*p*/*p*^o^) = (1.358 ± 0.005) ln(*t*_o_/*t*_a_)_avg_ − (2.74 ± 0.07) r^2^ = 0.9999						(25)
**9C**				**10^6^·*p*/Pa**	**10^6^·*p*/Pa**	
PMC	−8.407		−14.34 ± 0.27	60,000 ± 18,000		
n-Tetracosane	−13.948	−22.175	−22.13 ± 0.43	25 ± 8.6	24	
n-Octacosane	−17.101	−26.490	−26.57 ± 0.48	0.29 ± 0.11	32	
n-Triacontane	−18.650	−28.748	−28.75 ± 0.51	0.033 ± 0.013	0.033	
α-Tocopherol	−18.472		−28.50 ± 0.27	0.042 ± 0.017	0.008 ^d^	
n-Dotriacontane	−20.195	−30.964	−30.92 ± 0.54	0.0038 ± 0.0016	0.0036 ^e^	
ln(*p*/*p*^o^) = (1.407 ± 0.015) ln(*t*_o_/*t*_a_)_avg_ − (2.51 ± 0.27) r^2^ = 0.9998						(26)

^a^ Reference [20] unless noted otherwise. ^b^ Reference [21] unless noted otherwise. ^c^ Estimate, reference [9]. ^d^ Reported for the tocopherols [32]. ^e^ Reference [22].

**Table 10:** Constants of Equation (27) evaluated for PMC, Δ^9^-THC, and (±) α-TOC ^a^.

	A	B	C		
PMC				n-BP/K ^b^	
Runs 1 and 2	9.555 ± 0.113	−4119.6 ± 78	−889,762 ± 13,295		
Runs 3 and 4	9.682 ± 0.101	−4165.8 ± 70	−903,771 ± 11,943		
Runs 5 and 6	9.632 ± 0.102	−4185 ± 70.5	−883,491 ± 12,076		
Runs 1−6	9.631 ± 0.003	−4164.4 ± 0.2	−890,573 ± 33.8	589 ^c^	617 ± 42 ^d^
Δ^9^-THC				*p*_298.15 K_/Pa	
Runs 5 and 6	11.666 ± 0.128	−5670.6 ± 122.6	−1,296,183 ± 21,000	3.0·10^−5 c^	2.57·10^−5 e^
(±) α-TOC				*P*_549 K_/Pa	
Runs 7 and 8	13.502 ± 0.241	−7016.5 ± 166	−7016.5 ± 28,452	900 ^c^	1100 ^f^

^a^ Uncertainties represent one standard deviation; the value. ^b^ Normal boiling temperature. ^c^ This work. ^d^ Estimate, reference [35]. ^e^ Estimate, reference [9]. ^f^ References [32,33]; value for α-tocopherol.

## Data Availability

All relevant data is available in the Supplementary Materials.

## Supplementary Materials

The following material can be downloaded at https://www.mdpi.com/article/10.3390/molecules29184332/s1. Scheme S1. Adjustment of the vaporization enthalpy from *T*_m_ to *T* = 298.15 K of *trans*-bicyclo [4.3.0]nonane to *T* = 298.15 K; p. S1 (SM). Scheme S2 (A–F). Estimations of the vaporization enthalpy of PMC at *T* = 298.15 K; pp. S1–S2. Scheme S3. Estimates of the vaporization enthalpy of 5α-stigmastane via synthetic analysis; p. S10. Scheme S4. Conversion of PMC to “Δ^9^-THC” using n-butane; pg. S(11). Scheme S5. Estimations of the liquid heat capacity of Δ^9^-THC at *T* = 298.15 K; pg. S11. Vapor pressure comparisons for α-TOC. p. S12. Table S1. Experimental sublimation and fusion enthalpies and parameters for the temperature adjustment of both; p. S1. Table S2. Hydrocarbon group values used in the evaluation of the heat capacities of PMC and Δ^9^-THC in J.K^−1^.mol^−1^; p. S2. Table S3. Cyclic and acyclic functional group values including those used in evaluating heat capacities; pg S3. Tables S3A–S11A. Retention times of the standards and targets; pp. S4-S10. Tables S3B–S11B. Correlation between enthalpies of transfer and vaporization enthalpies; pp. S4-S11. Table S12. A comparison of vapor pressures for α TOC; p. S12. Figure S1. Gas chromatogram of PMC and Δ^9^-THC with alkane standards; p. S13. Figure S2. Gas chromatograph of PMC and α-TOC with alkane standards; p. S14. References p. S15 [37].
