# Supplementary material for: Estimation of the Vaporization Enthalpies and Vapor Pressures of α-Tocopherol and Δ9-Tetrahydrocannabinol via the Use of a Surrogate, Correlation Gas Chromatography, and Synthetic and Retrosynthetic Analysis
Source: Molecules. 2024 Sep 12;29(18):4332. doi: 10.3390/molecules29184332 (PMC11434254; doi:10.3390/molecules29184332)
Supplement: Supplementary file 1 [file molecules-29-04332-s001.zip › molecules-3074253-supplementary.pdf]

Supporting Material: Estimation of the vaporization enthalpies of (±) α-tocopherol and Δ^9-tetrahydrocannabinol by the use of a surrogate, correlation gas chromatography and synthetic and retrosynthetic analysis Carissa Nelson, Christian Fischer-Lodike and James S. Chickos

Department of Chemistry and Biochemistry
University of Missouri-St. Louis
St. Louis MO 63121 USA

Tetrahydrocannabinol (Δ^9-THC); 2,2,5,7,8-pentamethylchroman-6-ol (PMC); α-tocopherol (α-TOC)

Scheme S1. Adjustment of the vaporization enthalpy from T_m to T = 298.15 K of *trans*-bicyclo[4.3.0]nonane to T = 298.15 K

$\Delta_l{}^gH$(298.15 K)/kJ·mol^{-1} = $\Delta_l{}^gH$(T_m/K) + [10.58 + 0.26·Cp_l(298.15 K)]·[T_m -298.15]/1000 (S1)

trans bicyclo[4.3.0]nonane:
$\Delta_l{}^gH$(360 K) = 41.6 kJ·mol^{-1} [S2]: Cp_l(298.15 K = 214.2 J·mol^{-1}·K^{-1} [S3]

$\Delta_l{}^gH$(298.15 K)/kJ·mol^{-1} = $\Delta_l{}^gH$(T_m/K) + [10.58 + 0.26·Cp_l(298.15 K)]·[T_m -298.15]/1000 (S2)
$\Delta_l{}^gH$(298.15 K)/kJ·mol^{-1} = 41.6 + (4.0±0.8)
$\Delta_l{}^gH$(298.15 K)/kJ·mol^{-1} = 45.6±0.8

Table S1. Experimental sublimation and fusion enthalpies and parameters for the temperature adjustment of both

$\Delta_{cr}{}^gH$(341.5 K)	$\Delta_{cr}{}^lH(T_{fus})$ kJ·mol^{-1}	T_{fus}/K	$C_{p(cr)}/C_{p(l)}$ (298.15 K) J· K^{-1}·mol^{-1}	$\Delta C_p\Delta T$ kJ·mol^{-1}	$\Delta_{cr}{}^lH$(298.15 K) kJ·mol^{-1}
105.9±1.3 [a,b]	27.0±0.2 [b]	365.3 [b]	315.3 [b]/429.1[c]	-5.0±1.0 [d]	22.0±1.0

[a] Average of (105.8±1.6) and (105.9±0.9) kJ·mol^{-1} (mass effusion +Calvet drop calorimetry)

[b] Reference [S4]. [c] See Scheme 2E [S5]. [d] Uncertainty: 20 % of the temperature adjustment.

Scheme S2. Estimations of the vaporization enthalpy of PMC at *T* = 298.15 K

A. Adjustment of $\Delta_{cr}{}^lH(T_{fus})$ to $\Delta_{cr}{}^lH$(298.15 K) of PMC [S1]

$\Delta_{cr}{}^lH$(298.15 K) = $\Delta_{cr}{}^lH(T_{fus})$ + [0.15·$C_{p(cr)}$(298.15 K)- 0.26$C_{p(l)}$(298.15 K)-9.83)](ΔT) (S3)

$\Delta_{cr}{}^lH$(298.15 K) = (27.0±0.2)+[0.15·(315.3±9.6) - 0.26·429.1 - 9.83]·(365.3-298.15)/1000]

$\Delta_{cr}{}^lH$(298.15 K) =(**22.0±1.0**) kJ·mol^{-1}

B. Vaporization enthalpy at T = 298.15 K

$\Delta_l{}^gH$(298.15 K) = $\Delta_{cr}{}^gH$(298.15 K) - $\Delta_{cr}{}^lH$(298.15 K) (S4)

$\Delta_l{}^gH$(298.15 K) = (107.4±0.8) [S4] – (22.0±1.0) = **(85.4±1.3) kJ·mol^{-1}**

C. Adjustment of $\Delta_{cr}^{g}H$(341.5 K) to $\Delta_{cr}^{g}H$(365.3 K) of PMC; T_{fus} = 365.3 K

$\Delta_{cr}^{g}H$(341.5 K) = $\Delta_{cr}^{g}H$(341.5 K) + (0.75 + 0.15·315.3)·(341.45 - 365.3) (S5)

$\Delta_{cr}^{g}H$(341.5 K) = (105.9±1.3) - (1.1±0.2) = (**104.8±1.3**) **kJ·mol^{-1}**

D. Vaporization enthalpy at T_{fus}

$\Delta_{l}^{g}H(T_{fus}/K) = \Delta_{cr}^{g}H(T_{fus}) - \Delta_{cr}^{l}H(T_{fus})$

$\Delta_{l}^{g}H$(341.5 K) = (104.8±1.3) - (27.0±0.2) = (**77.8±1.3**) kJ·mol^{-1}

E. Estimation of the heat capacity of liquid PMC [**Tables S2, S3**]

$C_{p(cr)}$(298.15 K) = 5 CH_3- + 6 =C_a< + 2 >$C_{(c)}H_2$ + >$C_{(c)}$< + -OH_p + -$O_{(c)}$- (S6)

$C_{p(cr)}$(298.15 K) = 5·34.9 + 6·15.3 + 2·25.5 +13.9 + 74.9+ 23 = 429.1 J·K^{-1}·mol^{-1}

F. Adjustment of the vaporization enthalpy to T = 298.15 K

$\Delta_{l}^{g}H$(298.15 K) = (77.8±1.3) + (10.58 +0.26·429.1)·(365.3-298.15)/1000 **= 86.0±1.6 kJ·mol^{-1}**

Average: (85.5±1.3 + 86.0±1.6)/2 **= (85.8 ±1.5) kJ·mol^{-1}**

A check on the internal consistency of the results:

$\Delta_{cr}^{l}H$(298.15 K) + $\Delta_{l}^{g}H$(298.15 K) = $\Delta_{cr}^{g}H$(298.15 K) (S7)

(22.0±1.0) kJ·mol^{-1} + (85.8±1.5) kJ·mol^{-1} = (**107.8±1.8**) kJ·mol^{-1}; (107.4±0.8) [4]

Table S2. Hydrocarbon group values used in the evaluation of the heat capacities of PMC and Δ^9-THC in J·K^{-1}·mol^{-1} [S4]

**Hydrocarbon Functional Groups**	**Symbol**	$\Gamma_{(l)}$	$\Gamma_{(cr)}$	$\Gamma_{(l)}$ Previous
Primary sp^3 carbon	**CH_3-**	34.9	36.6	34.9
Secondary sp^3 carbon	**-CH_2-**	31.9	26.9	31.9
Tertiary sp^3 carbon	**>CH-**	22.4	9.0	22.4
Quaternary sp^3 carbon	**>C<**	14	-4.9	14
Secondary sp^{2}carbon	**=CH_2**	25.8	46	25.8
Tertiary sp^2 carbon	**=CH-**	27.8	21.4	27.8
Quaternary sp^2 carbon	**=C<**	21.7	6.9	21.7
**Cyclic Group Values**		$\Gamma_{(l)new}$[a]	$\Gamma_{(cr)}$	$\Gamma_{(l)}$ Previous
Cyclic secondary sp^3 carbon	**>$C_{(c)}H_2$**	**25.5**	24.6	25.9
Cyclic tertiary sp^3 carbon	**>$C_{(c)}$H-**	**17.4**	11.7	20.6
Cyclic quaternary sp^3 carbon	**>$C_{(c)}$<**	**13.9**	6.1	18
Cyclic tertiary sp^2 carbon	**=$C_{(c)}$H-**	**22.8**	18	21.8
Cyclic quaternary sp^2 carbon	**=$C_{(c)}$<**	**17.7**	6.6	21.2
**Aromatic Group Values Used** [S4]		$\Gamma_{(l)}$	$\Gamma_{(cr)}$	$\Gamma_{(l)}$ Previous
Tertiary aromatic sp^2 carbon	**=C_aH-**	21.8	17.5	21.8
Quaternary aromatic sp^2 carbon [b]	**=C_a<**	15.3	8.5	15.3

[a] All group values are based on more than 10 entries unless noted otherwise; values in bold are new or have been changed from previous versions. [b] Used for quaternary aromatic carbons in aromatic liquids.

Table S3. Cyclic and acyclic functional group values including those used in evaluating heat capacities by synthetic analysis; J·K^{-1}·mol^{-1} [S4]

**Cyclic Functional Groups**	**Symbol**	$\mathbf{\Gamma_{(l)\ new}}$[a]	$\mathbf{\Gamma_{(cr)}}$
Cyclic ether	**-O$_{(c)}$-**	**23**	10.3
**Monovalent Acyclic Groups**	**Symbol**	$\mathbf{\Gamma_{(l)}}$	$\mathbf{\Gamma_{(cr)}}$
Phenol	-OH$_p$	74.9 (2)	23.2

TABLE S4A.
Experimental retention times of PMC[a] and various n-alkanes on an HP-1 column

Run S1 T/K	452.7	457.9	462.9	468	473	478.1	483.2
t_o = 60 s				t/t_o			
CH_2Cl_2	0.936	0.949	0.951	0.962	0.967	0.974	0.979
n-Hexadecane	3.583	3.203	2.885	2.639	2.419	2.234	2.079
n-Heptadecane	4.900	4.294	3.795	3.401	3.061	2.777	2.541
PMC[a]	6.215	5.439	4.796	4.277	3.832	3.456	3.141
n-Nonadecane	9.783	8.295	7.062	6.106	5.303	4.641	4.101
n-Eicosane	14.053	11.721	9.842	8.365	7.155	6.165	5.361
n-Heneicosane	20.358	16.81	13.901	11.644	9.820	8.334	7.139

[a] 2,2,5,7,8-pentamethylchroman-6-ol.

TABLE S4B.
Enthalpies of Transfer and Vaporization Enthalpies Used to Evaluate Vaporization Enthalpies of PMC (p^o/Pa =101325)[a]

Run S1	- slope<br>T/K	intercept	ΔH_{trn}(468 K)<br>kJ·mol^{-1}	$\Delta_l{}^gH$(298 K)<br>kJ·mol^{-1} (lit)[b]	$\Delta_l{}^gH$(298 K)<br>kJ·mol^{-1}(calc)
n-Hexadecane	6293.4±22	12.933±0.047	52.32±0.18	81.35±0.8	81.4±1.1
n-Heptadecane	6680.9±22	13.385±0.047	55.54±0.18	86.47±1.7	86.3±1.1
PMC	6405.4±18	12.489±0.039	53.25±0.15		82.8±1.1
n-Nonadecane	7484.9±25	14.357±0.054	62.23±0.21	96.44±1.9	96.7±1.2
n-Eicosane	7876.3±25	14.828±0.053	65.48±0.20	101.81±2.0	101.8±1.2
n-Heneicosane	8259.4±0.27	15.28±0.057	68.67±0.22	106.8±2.2 [c]	106.7±1.2

$\Delta_l{}^gH$(298.15 K)/kJ·mol^{-1} = (1.55±0.01 4)ΔH_{trn}(468 K) + (0.21±0.0.83) r^2 = 0.9998 (S8)

[a] Uncertainties represent one standard deviation unless noted otherwise. [b] Reference [S5] unless noted otherwise; uncertainties represent probable error. [c] Reference [S6].

TABLE S5A.

Experimental Retention Times of PMC[a] and Various n-Alkanes on a 12 m HP-1 column

Run S2 T/K	452.7	457.9	463	468	473.1	478.2	483.2
t_o = 60 s				t/t_o			
CH_2Cl_2	0.937	0.947	0.955	0.964	0.972	0.961	0.986
n-Hexadecane	3.580	3.197	2.892	2.635	2.424	2.226	2.089
n-Heptadecane	4.902	4.288	3.802	3.399	3.068	2.771	2.552
PMC[a]	6.222	5.433	4.803	4.277	3.840	3.453	3.154
n-Nonadecane	9.817	8.287	7.084	6.103	5.315	4.644	4.117
n-Eicosane	14.105	11.718	9.861	8.369	7.173	6.174	5.380
n-Heneicosane	20.512	16.807	13.942	11.655	9.844	8.352	7.163

[a] 2,2,5,7,8-Pentamethylchroman-6-ol

TABLE S5B.

Enthalpies of Transfer and Vaporization Enthalpies Used to Evaluate Vaporization Enthalpies of PMC (p^o/Pa =101325)[a]

Run S2	- slope T/K	intercept	ΔH_{trn}(468 K) kJ·mol^{-1}	$\Delta_l^g H$(298 K) kJ·mol^{-1} (lit)[b]	$\Delta_l^g H$(298 K) kJ·mol^{-1}(calc)
n-Hexadecane	6250.6±20	12.840±0.043	51.96±0.17	81.35±0.8	81.4±1.1
n-Heptadecane	6646.1±29	13.309±0.049	55.25±0.19	86.47±1.7	86.4±1.1
PMC[a]	6374.6±20	12.421±0.044	53.00±0.17		82.9±1.1
n-Nonadecane	7464.6±24	14.310±0.051	62.06±0.20	96.44±1.9	96.7±1.2
n-Eicosane	7856.9±25	14.783±0.053	65.32±0.20	101.81±2.0	101.7±1.2
n-Heneicosane	8261.1±25	15.279±0.053	68.68±0.21	106.8±2.2 [c]	106.8±1.2

$\Delta_l^g H$(298.15 K)/kJ·mol^{-1} = (1.52±0.01 3)ΔH_{trn}(468 K) + (2.35±0.0.82) r^2 = 0.9998   (S9)

[a] Uncertainties represent one standard deviation unless noted otherwise. [b] Reference [S5] unless noted otherwise; uncertainties represent probable error. [c] Reference [S6].

TABLE S6A.

Experimental Retention Times of PMC, THC and Various n-Alkanes on a 12 m HP-1 column[a]

Run S3 t_o = 60 s	T/K	488.1	493.1	498.2	503.2	508.3	513.4	518.4
					t/t_o			
CH_2Cl_2		0.984	0.989	0.998	1.005	1.001	1.014	1.014
n-Eicosane		4.719	4.174	3.726	3.356	3.026	2.775	2.549
n-Heneicosane		6.180	5.383	4.731	4.198	3.731	3.370	3.055
n-Docosane		8.190	7.038	6.098	5.331	4.675	4.160	3.722
n-Tetracosane		14.773	12.376	10.453	8.909	7.609	6.594	5.752
Δ^9-THC [b]		17.989	15.073	12.783	10.932	9.320	8.099	7.061
n-Pentacosane		20.032	16.548	13.851	11.662	9.839	8.436	7.267
n-Hexacosane		27.18	22.304	18.417	15.346	12.800	10.854	9.274

[a] (6aR,10aR)-Δ-9-Tetrahydrocannabinol

TABLE S6B

Enthalpies of Transfer and Vaporization Enthalpies Used to Evaluate Vaporization Enthalpies of PMC and THC [a]

Run S3	- slope T/K	intercept	ΔH_{trn}(503 K) kJ·mol^{-1}	Δ_l^gH(298 K) kJ·mol^{-1} (lit)[b]	Δ_l^gH(298 K) kJ·mol^{-1}(calc)
n-Eicosane	-7416.8±33	13.883±0.065	61.66±0.27	101.81±2.0 [c]	101.8±0.3
n-Heneicosane	-7795.4±36	14.329±0.071	64.81±0.30	106.8±2.2	106.9±0.3
n-Docosane	-8169.2±35	14.768±0.070	67.92±0.29	111.9±2.7	111.9±0.3
n-Tetracosane	-8916.6±38	15.651±0.076	74.13±0.32	121.9±2.8	121.9±0.3
Δ^9-THC	-8621.8±47	14.839±0.076	71.68±0.39		117.9±0.3
n-Pentacosane	-9286.7±46	16.088±0.091	77.21±0.38	121.9±2.9	126.8±0.3
n-Hexacosane	-9647.7±49	16.508±0.098	80.21±0.41	126.8±3.2	131.7±0.3

$$\Delta_l^gH(298.15\ \text{K})/\text{kJ·mol}^{-1} = (1.612\pm0.00[illegible])\Delta H_{trn}(503\ \text{K}) + (2.396\pm0.190) \qquad r^2 = 0.9999 \qquad \text{(S10)}$$

[a] Uncertainties represent one standard deviation unless noted otherwise. [b]Reference [S6] unless noted otherwise. [c] Reference [S5]; uncertainty represents probable error.

TABLE S7A.

Experimental Retention Times of PMC, THC and Various n-Alkanes on a 12 m HP-1 column[a]

Run S4	*T*/K	488	493.1	498.1	503.2	508.3	513.4	518.2
t_o = 60 s					t/t_o			
CH_2Cl_2		0.974	0.979	0.982	0.987	0.991	0.995	0.998
n-Eicosane		4.656	4.12	3.666	3.296	2.982	2.725	2.507
n-Heneicosane		6.095	5.311	4.658	4.124	3.678	3.313	3.008
n-Docosane		8.085	6.946	6.005	5.24	4.607	4.092	3.666
n-Tetracosane		14.583	12.228	10.298	8.764	7.508	6.494	5.672
Δ^9-THC [b]		17.78	14.946	12.631	10.758	9.214	7.981	6.965
n-Pentacosane		19.773	16.385	13.651	11.48	9.717	8.31	7.171
n-Hexacosane		26.848	22.001	18.139	15.097	12.638	10.695	9.128

[a] (6aR,10aR)-Δ-9-Tetrahydrocannabinol

TABLE S7B

Enthalpies of Transfer and Vaporization Enthalpies Used to Evaluate Vaporization Enthalpies of PMC and THC [a]

Run S4	- slope *T*/K	intercept	ΔH_{trn}(503 K) kJ·mol^{-1}	$\Delta_l^g H$(298 K) kJ·mol^{-1} (lit)[b]	$\Delta_l^g H$(298 K) kJ·mol^{-1}(calc)
n-Eicosane	-7455.8±28	13.979±0.056	61.98±0.23	101.81±2.0 [c]	101.9±0.4
n-Heneicosane	-7820.2±30	14.396±0.59	65.01±0.25	106.8±2.2	106.8±0.4
n-Docosane	-8195.7±32	14.838±0.064	68.14±0.27	111.9±2.7	111.8±0.4
n-Tetracosane	-8936.6±33	15.707±00.65	74.30±0.27	121.9±2.8	121.8±0.4
Δ^9-THC	-8660.3±32	14.930±0.064	72.00±0.27		118.1±0.4
n-Pentacosane	-9309.1±34	16.148±0.067	77.39±0.28	121.9±2.9	126.8±0.4
n-Hexacosane	-9673.3±37	16.575±0.074	80.42±0.31	126.8±3.2	131.7±0.4

$\Delta_l^g H$(298.15 K)/kJ·mol^{-1} = (1.619±0.004)ΔH_{trn}(503 K) + (1.47±0.29) r^2 = 0.9999   (S11)

[a] Uncertainties represent one standard deviation unless noted otherwise. [b] Reference [S6] unless noted otherwise. [c] Reference [S5]; uncertainty represents probable error.

TABLE S8A.

Experimental Retention Times of PMC, THC and Various n-Alkanes on a 12 m HP-1 column

Run S5	T/K	488.2	493.3	498.3	503.4	508.6	513.6	518.6
t_o = 60 s					t/t_o			
CH_2Cl_2		0.989	0.994	0.997	0.997	1.009	1.012	1.014
PMC[a]		2.895	2.673	2.467	2.295	2.166	2.037	1.930
n-Eicosane		4.727	4.185	3.722	3.338	3.037	2.767	2.545
n-Heneicosane		6.183	5.392	4.725	4.178	3.741	3.362	3.051
n-Docosane		8.199	7.046	6.088	5.310	4.684	4.148	3.712
n-Tetracosane		14.789	12.388	10.434	8.875	7.622	6.577	5.731
Δ^9-THC [b]		17.915	15.067	12.712	10.839	9.318	8.051	7.020
n-Pentacosane		20.018	16.593	13.813	11.616	9.858	8.410	7.239
n-Hexacosane		27.180	22.289	18.354	15.280	12.825	10.826	9.222

[a] 2,2,5,7,8-Pentamethylchroman-6-ol

[b] (6aR,10aR)-Δ-9-Tetrahydrocannabinol

TABLE S8B.

Enthalpies of transfer and vaporization enthalpies used to evaluate the vaporization enthalpies of PMC and THC; (p^o/Pa =101325)[a]

Run S5	- slope T/K	intercept	ΔH_{trn}(503 K) kJ·mol^{-1}	$\Delta_l^g H$(298 K) kJ·mol^{-1} (lit)[b]	$\Delta_l^g H$(298 K) kJ·mol^{-1}(calc)[c]
PMC	6102.8±41	11.858±0.081	50.74±0.34		84.1±0.4
n-Eicosane	7426.9±37	13.898±0.074	61.74±0.31	101.81±2.0 [c]	101.5±0.4
n-Heneicosane	7790.2±36	14.314±0.072	64.76±0.30	106.8±2.2	106.4±0.4
n-Docosane	8178.0±35	14.781±0.071	67.99±0.30	111.9±2.7	111.7±0.4
n-Tetracosane	8927.0±37	15.667±0.074	74.22±0.31	121.9±2.8	122.0±0.4
Δ^9-THC	8620.8±40	14.835±0.080	71.67±0.33		117.8±0.4
n-Pentacosane	9292.8±38	16.095±0.075	77.26±0.32	126.8±2.9	127.0±0.4
n-Hexacosane	9649.0±42	16.506±0.83	80.22±0.35	131.7±3.2	131.9±0.5

$$\Delta_l^g H(298.15\ \text{K})/\text{kJ·mol}^{-1} = (1.612±0.004)\Delta H_{trn}(503\ \text{K}) + (2.306±0.33) \qquad r^2 = 0.9997 \quad \text{(S12)}$$

[a] Uncertainties represent one standard deviation unless noted otherwise. [b] Reference [S6] unless noted otherwise. [c] Reference [S5]; uncertainty represents probable error.

TABLE S9A.

Experimental Retention Times of PMC, THC and Various n-Alkanes on a 12 m HP-1 column[a]

Run S6 t_o = 60 s	T/K	488	493.3	498.4	503.4	508.5	513.5	518.5
					t/t_o			
CH_2Cl_2		0.976	0.983	0.988	0.993	0.997	1.001	0.998
PMC[a]		2.866	2.640	2.444	2.284	2.138	2.019	1.906
n-Eicosane		4.680	4.139	3.688	3.319	3.000	2.741	2.516
n-Heneicosane		6.125	5.338	4.683	4.150	3.696	3.330	3.016
n-Docosane		8.125	6.976	6.034	5.272	4.628	4.111	3.675
n-Tetracosane		14.646	12.276	10.354	8.804	7.534	6.524	5.682
Δ^9-THC [b]		17.730	14.923	12.619	10.766	9.222	7.992	6.960
n-Pentacosane		19.860	16.438	13.708	11.527	9.744	8.344	7.183
n-Hexacosane		26.955	22.100	18.219	15.169	12.689	10.740	9.131

[a] 2,2,5,7,8-Pentamethylchroman-6-ol. [b] (6aR,10aR)-Δ-9-Tetrahydrocannabinol

TABLE S9B

Enthalpies of Transfer and Vaporization Enthalpies Used to Evaluate Vaporization Enthalpies of PMC and THC [a]

Run S6	- slope T/K	intercept	ΔH_{trn}(503 K) kJ·mol^{-1}	$\Delta_l^g H$(298 K) kJ·mol^{-1} (lit)[b]	$\Delta_l^g H$(298 K) kJ·mol^{-1}(calc)
PMC	6123.6±16	11.910±0.033	59.91±0.13		84.0±0.3
n-Eicosane	7457.8±22	13.971±0.044	62.00±0.18	101.81±2.0 [c]	101.8±0.3
n-Heneicosane	7834.0±24	14.412±0.047	65.13±0.20	106.8±2.2	106.8±0.3
n-Docosane	8214.6±27	14.864±0.053	68.29±0.22	111.9±2.7	111.9±0.3
n-Tetracosane	8958.9±26	15.740±0.051	74.48±0.22	121.9±2.8	121.8±0.3
Δ^9-THC	8642.5±23	14.888±0.046	71.85±0.25		117.6±0.3
n-Pentacosane	9331.1±30	16.180±0.059	77.58±0.25	126.8±2.9	126.8±0.3
n-Hexacosane	9704.2±24	16.625±0.047	80.68±0.20	131.8±3.2	131.8±0.3

$$\Delta_l^g H(298.15\ K)/kJ\cdot mol^{-1} = (1.603\pm0.00[illegible])\Delta H_{trn}(503\ K) + (2.399\pm0.220) \quad r^2 = 0.9999 \quad (S13)$$

[a] Uncertainties represent one standard deviation unless noted otherwise. [b] Reference [S6] unless noted otherwise. [c] Reference [S5; uncertainty represents probable error.

TABLE S10A.

Experimental Retention Times of (±) α-Tocopherol[a] and Various n-Alkanes on an HP-1 Column

Run S7 t_o = 60 s	T/K	518.5	523.6	528.6	533.6	538.5	543.3	548
					t/t_o			
CH_2Cl_2		1.031	1.034	1.039	1.044	1.045	1.051	1.056
PMC[a]		1.947	1.857	1.778	1.707	1.648	1.594	1.549
n-Tetracosane		5.787	5.084	4.504	4.012	3.606	3.269	2.985
n-Octacosane		15.33	12.899	10.926	9.302	7.992	6.935	6.055
n-Triacontane		25.558	21.132	17.553	14.681	12.374	10.544	9.024
(±)α-Tocopherol		31.883	26.398	22.032	18.497	15.551	13.295	11.389
n-Dotriacontane		42.957	35.011	28.584	23.523	19.485	16.315	13.732

[a] 2,2,5,7,8-pentamethylchroman-6-ol

TABLE S10B.

Enthalpies of Transfer and Vaporization Enthalpies Used to Evaluate Vaporization Enthalpies of (±α-Tocopherol (p^o/Pa =101325)[a]

Run S7	- slope T/K	intercept	ΔH_{trn}(533 K) kJ·mol^{-1}	Δ_l^gH(298 K) kJ·mol^{-1} (lit)[b]	Δ_l^gH(298 K) kJ·mol^{-1}(calc)
PMC	5961.2±30	11.582±0.057	49.56±0.25		82.7±1.7
n-Tetracosane	8688.8±18	15.198±0.034	72.24±0.15	121.9±2.8	121.7±1.9
n-Octacosane	10122.8±21	16.862±0.039	84.16±0.17	141.9±4.9	142.2±2.0
n-Triacontane	10827.4±23	17.682±0.043	90.01±0.19	152.3±0.6	152.3±2.1
(±) α-Tocopherol	10533.7±25	16.886±0.047	87.57±0.21		148.1±2.0
n-Dotriacontane	11528.2±29	18.496±0.055	95.84±0.24	162.5±0.7[c]	162.3±2.1

$$\Delta_l^gH(298.15\text{ K})/\text{kJ·mol}^{-1} = (1.720\pm0.0(\; 7)\Delta H_{trn}(533\text{ K}) - (2.486\pm1.484) \qquad r^2 = 0.9999 \qquad (S14)$$

[a] Uncertainties represent one standard deviation; references from ref. [S6] unless noted otherwise. c Reference [S

TABLE S11A.

Experimental Retention Times of PMC and (±) α-Tocopherol[a] and Various n-Alkanes on a 12 m HP-1 column

Run S8	T/K	518.5	523.6	528.7	533.7	538.5	543.2	547.7
t_o = 60 s					t/t_o			
CH_2Cl_2		1.035	1.04	1.042	1.048	1.051	1.056	1.068
PMC[a]		1.957	1.867	1.784	1.716	1.653	1.603	1.570
n-Tetracosane		5.824	5.117	4.523	4.031	3.616	3.292	3.035
Octacosane		15.434	12.983	10.972	9.353	8.015	6.982	6.164
n-Triacontane		25.751	21.278	17.640	14.766	12.426	10.616	9.194
(±) α-Tocopherol		32.175	26.707	22.204	18.641	15.662	13.426	11.604
n-Dotriacontane		43.303	35.243	28.720	23.644	19.560	16.434	13.984

[a] 2,2,5,7,8-pentamethylchroman-6-ol

TABLE S11B.

Enthalpies of Transfer and Vaporization Enthalpies Used to Evaluate Vaporization Enthalpies of PMC and (±) α-Tocopherol (p^o/Pa =101325)[a]

Run S8	- slope T/K	intercept	ΔH_{trn}(533 K) kJ·mol^{-1}	Δ_l^gH(298 K) kJ·mol^{-1} (lit)	Δ_l^gH(298 K) kJ·mol^{-1}(calc)
PMC	5951.0±28	11.557±0.052	49.47±0.23		83.0±1.4
n-Tetracosane	8688.8±29	15.191±0.055	72.24±0.24	121.7±2.8	121.8±1.6
n-Octacosane	10133.2±32	16.875±0.060	84.24±0.27	141.9±4.9	142.2±1.7
n-Triacontane	10845.3±31	17.708±0.058	90.16±0.26	152.3±0.6	152.3±1.8
(±) α-Tocopherol	10566.6±42	16.938±0.078	87.85±0.35		148.3±1.8
n-Dotriacontane	11557.6±36	18.544±0.068	96.09±0.30	162.5±0.7 [b]	162.4±1.8

Δ_l^gH(298.15 K)/kJ·mol^{-1} = (1.703±0.014)ΔH_{trn}(533 K) - (1.234±1.246) r^2 = 0.9999   (S15)

[a] Uncertainties represent one standard deviation unless noted otherwise; ref. [S2] unless noted otherwise [b] Reference [S3].

Scheme S3. Estimates of the vaporization enthalpy of 5α-stigmastane by synthetic analysis

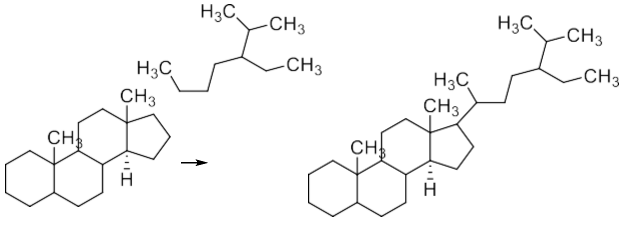

Androstane: Δ_l^gH(298 K) = (85.4±0.9(kJ·mol^{-1}

2-methyl-3-ethylheptane: 48.1 kJ·mol^{-1} [S8]

5α-stigmastane: Δ_l^gH(298 K) = (85.4±0.9) + (48.1±0.2) + 2·[CH(C)$_3$ - CH$_2$(C)$_2$]   (S16)

Δ_l^gH(298 K) = (85.4±0.9) + 48.1+ 2·[(3.05±0.13) - (5.06±0.04)] = (129.5 ±0.9) kJ·mol^{-1}

Scheme S4. Conversion of PMC to "Δ^9-THC" using n-butane

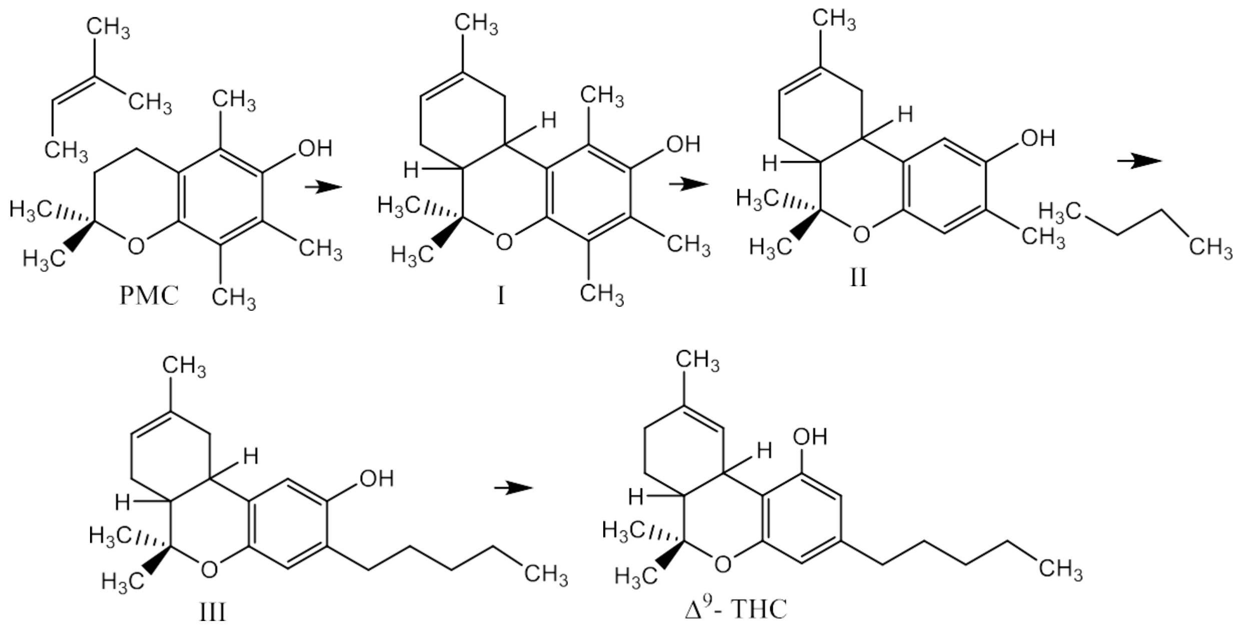

Step 2: Demethylation

$\Delta_l^g H$(298.15 K)/kJ·mol^{-1} = (108.3±2.6) + 2·[C_B(H) - C_B(C) - (CH_3(C)] (S17)

$\Delta_l^g H$(298.15 K)/kJ·mol^{-1} = (108.3±2.6) + 2·[(5.61±0.08) - (4.64±0.21) - (5.69±0.04)]

$\Delta_l^g H$(298.15 K)/kJ·mol^{-1} = (98.9 ±2.7)

Step 3: Alkylation

$\Delta^{lg} H$(298.15 K)/kJ·mol^{-1} = (98.9±2.7) + (22.4±0.1) + [(CH_2(C)C_B) + (CH_2(C_2) - 2(CH_3(C)] (S18)

$\Delta_l^g H$(298.15 K)/kJ·mol^{-1} = (98.9±2.7) + (22.4±0.1) + [(3.31±0.75)+(5.06±0.04) - 2·(5.69±0.04)]

$\Delta_l^g H$(298.15 K)/kJ·mol^{-1} = (118.3 ±2.8) kJ·mol^{-1}.

Scheme S5. Estimation of the liquid heat capacity of Δ^9-THC at T = 298.15 K (Tables S2, S3)

$C_{p(l)}$(298.15 K) = 4 CH_3- + 4-CH_2- + 4 =C_a< + 2 =C_aH- + 2 >$C_{(c)}H_2$ + 2 >$C_{(c)}$H- + >$C_{(c)}$< +
=C_cH- + =C_c< + -OH_p + -$O_{(c)}$- (S19)

$C_{p(l)}$(298.15 K) = 4·34.9 + 4·31.9 + 4·15.3 + 2·21.8 + 2·25.5 + 2·17.4 + 13.9 + 22.8 +17.7 +
74.9 + 23 = 610.1 J·K^{-1}·mol^{-1}

Table S12. A comparison of the vapor pressures of α-TOC evaluated in this work (Eq. (S20)) with literature values calculated using Eq. (S21)

T/K	p/Pa This work	p/Pa [S9]
298.15	$42\cdot10^{-9}$	$7.9\cdot10^{-9}$
310	$41\cdot10^{-8}$	$8.4\cdot10^{-8}$
320	$24\cdot10^{-7}$	$5.3\cdot10^{-7}$
330	$12\cdot10^{-6}$	$3.0\cdot10^{-6}$
340	$5.5\cdot10^{-5}$	$1.5\cdot10^{-5}$
350	$22\cdot10^{-5}$	$6.6\cdot10^{-5}$
360	$8.0\cdot10^{-4}$	$2.7\cdot10^{-4}$
370	$2.7\cdot10^{-3}$	$1.0\cdot10^{-3}$
380	$8.2\cdot10^{-3}$	$3.4\cdot10^{-3}$
390	$2.3\cdot10^{-2}$	$1.1\cdot10^{-2}$
400	$6.2\cdot10^{-2}$	$3.3\cdot10^{-2}$
410	0.16	0.092
420	0.37	0.24
430	0.84	0.60
440	1.8	1.4
450	3.8	3.2
460	8.0	7.0
470	14	14
480	27	29
490	48	55
500	84	100

Results rounded to two significant figures

$\ln(p/p^{o}) = 13.502 - 7016.5/(T/\mathrm{K}) - 1642332/(T/\mathrm{K})^2$; $p^{o} = 101325$; Pa this work. $^{+}$ (S20)

$\ln(p/\mathrm{Pa}) = 50.449 - 20228.9/(T) + 0.9786\cdot\ln(T/\mathrm{K}) - 0.0229\cdot T^{1}$ [S9] (S21)

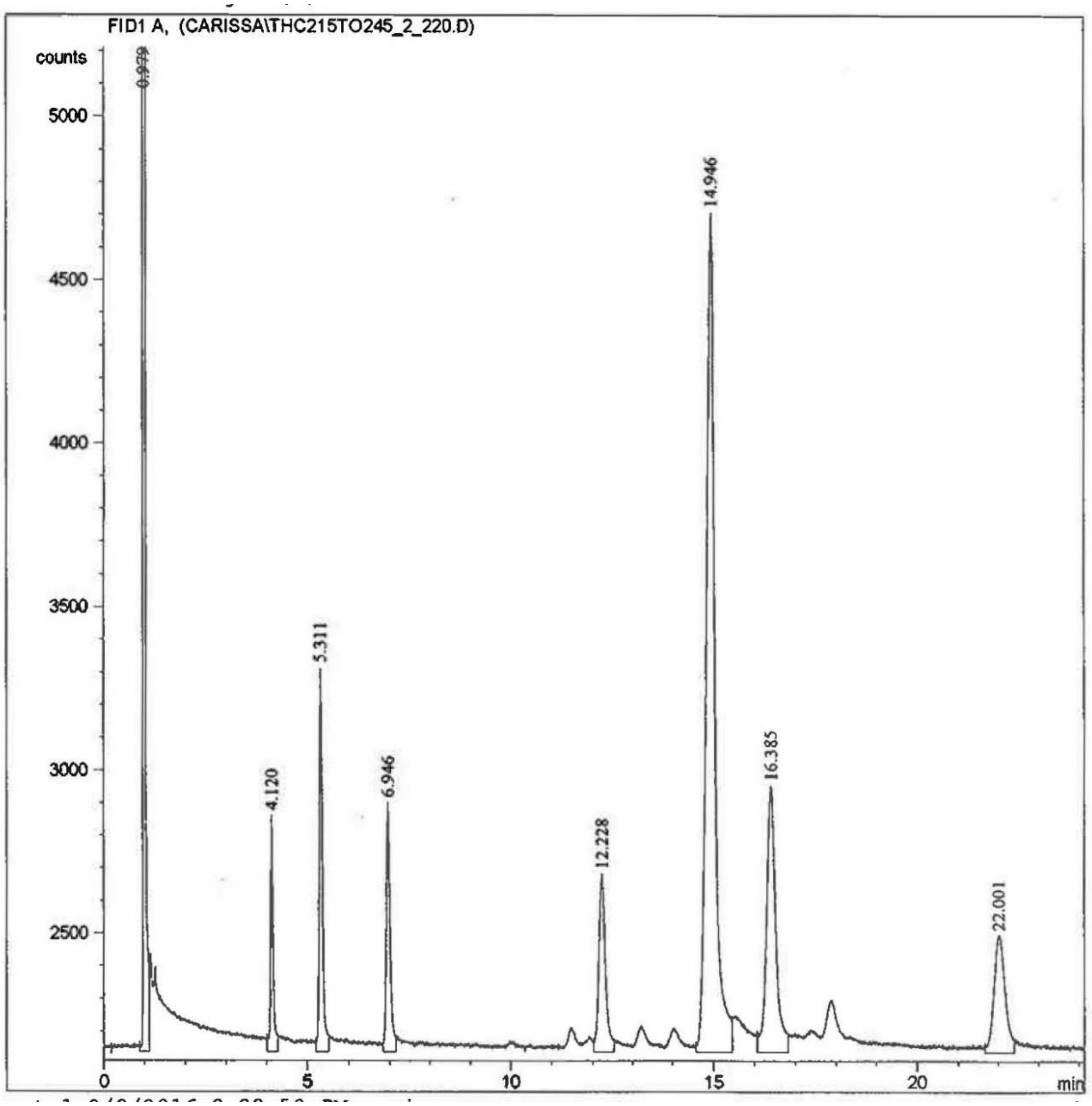

Figure S1. Gas chromatography of PMC and Δ^9-THC with alkane standards at T = 219.9 °C on a 12 m HP 1 column. Retention times t/60: 0.979, solvents CH_2Cl , methanol; 4.120, n-eicosane; 5. 311, n-heneicosane; 8.085, n-docosane; 6.946, n-tetracosane; 12.228, Δ^9-THC; 14.946, n-pentacosane; 16.385 n-hexacosane, 22.001.

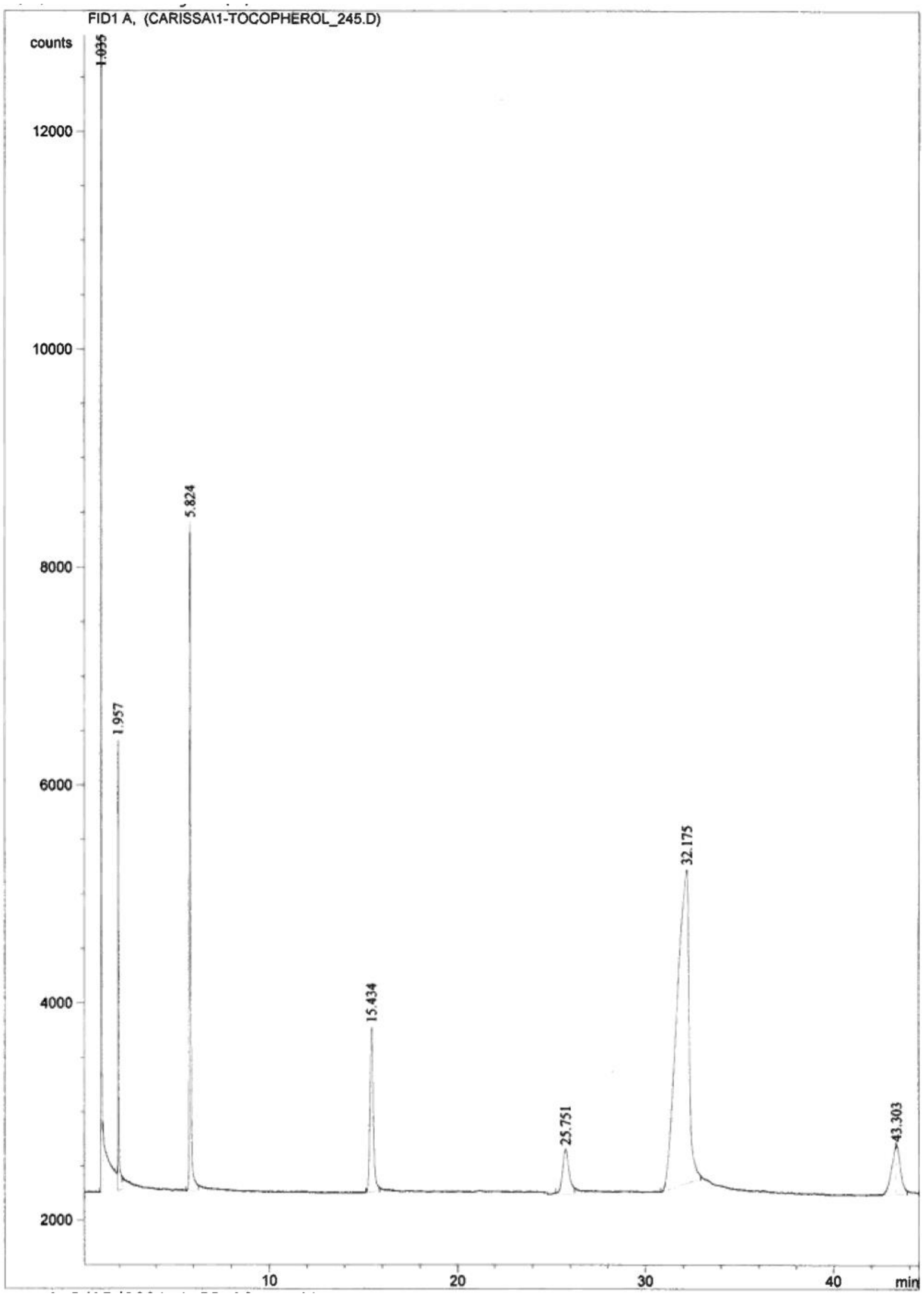

Figure S2. Gas chromatography of PMC and α-TOC with alkane standards at T = 245.3 °C on a 12 m HP 1 column. Retention times t/60: 1.035, CH_2Cl_2; 1.957, PMC; 5.824, n-tetracosane; 15.434, n-octacosane; 25.751, n-triacontane; 32.175, α-TOC; 43.303, n-dotriacontane.

References

[S1] Acree, Jr. W., Chickos, J. S. Phase Transition Enthalpy Measurements of Organic and Organometallic Compounds. Sublimation, Vaporization and Fusion Enthalpies From 1880 to 2015. Part 1. $C_1 - C_{10}$. *J. Phys. Chem. Ref. Data* **2016,** *45*, 033101.

[S2] Camin D. L.; Rossini, F. D. Physical Properties of 14 American Petroleum Institute Research Hydrocarbons, C_9 to C_{15}. *J. Phys. Chem*. **1955**, *59*,1173–1179.

[S3] Zabransky, M.; Ruzicka Jr., V.; Majer, V.; Domalski, E. S. Heat capacity of liquids, Vol 1. Critical Review and Recommended Values. *J. Phys. Chem. Ref. Data*, Monograph No. 6 1996.

[S4] Bernardes, C. E. S. ; Simoes, R. G.; Diogo, H. P.; Minas da Piedade, M. E. Thermochemistry of 2,2,5,7,8-pentamethylchroman-6-ol (PMC) and 6-hydroxy-2,5,7,8-tetramethylchroman-2-carboxylic acid (trolox*). J. Chem. Thermodyn*. **2014**, *73,*140-147.

[S5] Chickos, J. An update on liquid heat capacity estimations of cyclic organic compounds by group additivity and their application in estimations of complex molecules by synthetic and retrosynthetic analysis. *J. Chem. Thermodyn*. **2023**, *182*, 107039, 1-12.

[S5] Ruzicka, K.; Majer, V. Simultaneous Treatment of Vapor Pressures and Related Thermal Data Between the Triple and Normal Boiling Temperatures for n-Alkanes C_5 - C_{20}. *J. Phys. Chem. Ref. Data* **1994**, *23*, 1-39

[S6] Chickos, J. S.; Hanshaw, W. Vapor Pressures and Vaporization Enthalpies of the *n*-Alkanes from C_{21} to C_{30} at T = 298.15 K by Correlation Gas Chromatography *J. Chem. Eng. Data* **2004**, *49*, 77-85.

[S7] Chickos, J. S.; Hanshaw, W. Vapor Pressures and Vaporization Enthalpies of the *n*-Alkanes from C_{31} to C_{38} at T = 298.15 K by Correlation Gas Chromatography *J. Chem. Eng. Data* **2004**, *49*, 620-630.

[S8] Wilhoit R. C. ; Zwolinski, B. J. “Handbook of vapor pressures and heats of vaporization of hydrocarbons and related compounds,” in API 44-TRC Publications in Science and Engineering (API, College Station, TX, 1971)

[S9] Kerkache, H.; Bathily, A.; Chiriac, R.; Goutaudier, C.; Paricaud1, P.; Nicolle, A. Vapor-Liquid equilibria of α- tocopherol in transportation fuels surrogates: an experimental and modeling study. *Fuel* 2022, 319, 123866.
